# TRIM56 Modulates YBX1 Degradation to Ameliorate ZBP1‐Mediated Neuronal PANoptosis in Spinal Cord Injury

**DOI:** 10.1002/advs.202407132

**Published:** 2024-09-18

**Authors:** Junsheng Lou, Yiting Mao, Wu Jiang, Honghao Shen, Yunpeng Fan, Qing Yu, Conghui Zhou, Ziyao Wei, Kailiang Zhou, Mengran Jin, Junsong Wu

**Affiliations:** ^1^ Department of Orthopedic Surgery The First Affiliated Hospital Zhejiang University School of Medicine No. 79 Qingchun Road Hangzhou 310003 China; ^2^ Obstetrics and Gynecology Hospital Institute of Reproduction and Development Fudan University Shanghai 200090 China; ^3^ Department of Orthopeadics Affiliated Hangzhou First People's Hospital School of Medicine Westlake University No. 261 Huansha Road Hangzhou 310006 China; ^4^ Department of Critical Care Medicine the Second Affiliated Hospital of Zhejiang University School of Medicine Hangzhou 310009 China; ^5^ Department of Orthopeadics The Second Affiliated Hospital and Yuying Children's Hospital of Wenzhou Medical University Wenzhou 325027 China

**Keywords:** PANoptosis, spinal cord injury, TRIM56, YBX1, ZBP1

## Abstract

Spinal cord injury (SCI) is a severe injury to the central nervous system, and its treatment is always a major medical challenge. Proinflammatory cell death is considered an important factor affecting neuroinflammation and the prognosis after injury. PANoptosis, a newly discovered type of proinflammatory cell death, regulates the activation of executioner molecules of apoptosis, pyroptosis and necroptosis through the PANoptosome, providing a new target for therapeutic intervention after SCI. However, its role and regulatory mechanism in SCI are not yet elucidated. Here, based on proteomic data, YBX1 expression is significantly increased in neurons after SCI. Guided by RIP‐seq, subsequent experiments reveal that YBX1 promotes ZBP1 expression by stabilizing the *Zbp1* mRNA, thereby aggravating ZBP1‐mediated PANoptosis. Furthermore, the E3 ubiquitin ligase TRIM56 is identified as an endogenous inhibitor of YBX1 via molecular docking and IP/MS analysis. Mechanistically, TRIM56 bound to YBX1 and promoted its ubiquitination, thereby accelerating its degradation. Taken together, these findings reveal a novel function of YBX1 in regulating ZBP1‐mediated PANoptosis in the pathogenesis of SCI and verified that TRIM56 functions as an endogenous inhibitor to promote the ubiquitin‐proteasomal degradation of YBX1, providing new insights into SCI treatment strategies.

## Graphical abstract

Spinal cord injury (SCI) is a severe injury to the central nervous system, and its treatment is always a major medical challenge. The current study reveals a novel function of YBX1 in regulating ZBP1‐mediated PANoptosis in the pathogenesis of SCI and verified that TRIM56 functions as an endogenous inhibitor to promote the ubiquitin‐proteasomal degradation of YBX1, providing new insights into SCI treatment strategies.

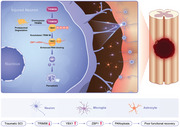

## 1 Introduction

Spinal cord injury (SCI) is a severe injury to the central nervous system. Millions of patients worldwide suffer from SCI and its neurological sequelae and complications, such as respiratory disorders, renal failure, cardiac dysfunction, and infections from untreated pressure ulcers.^[^ ^1^ ^]^ In traumatic SCI, the primary injury damages cells and subsequently initiates a complex secondary injury.^[^ ^2^ ^]^ Periodic inflammation and cell death during the secondary injury phase result in the massive loss of neurons and glial cells, resulting in the poor intrinsic recovery potential of the spinal cord.^[^ ^3^ ^]^ At present, no effective treatment has been developed and successfully translated for clinical application. Considering the inevitability of primary injury, positive intervention in the early stages of secondary injury to protect neurons is a widely accepted strategy to improve neurological function.

Neuroinflammation and proinflammatory cell death are important challenges after SCI. Inflammation is an important protective process in the body.^[^ ^4^ ^]^ However, eliminating inflammatory stimuli in time during the secondary injury stage is difficult, resulting in persistent inflammation and proinflammatory cell death.^[^ ^5^ ^]^ The occurrence of proinflammatory cell death provides a new material basis for the continuation of inflammation, causing inflammation and cell death to cycle back and forth and ultimately leading to a poor prognosis. Therefore, disrupting this pathological process has important practical importance for functional recovery after SCI. Many types of proinflammatory cell death have been discovered, and most studies have focused on only one type of death.^[^ ^6^ ^]^ PANoptosis is a new type of proinflammatory cell death established in recent years based on the extensive crosstalk among apoptosis, pyroptosis and necroptosis.^[^ ^7^ ^]^ Previous studies have shown that the formation of the PANoptosome is crucial for the process of PANoptosis.^[^ ^8^ ^]^ The PANoptosome is a multiprotein complex that acts as a molecular scaffold for the simultaneous participation of key molecules in pyroptosis, apoptosis, and necroptosis.^[^ ^9^ ^]^ Furthermore, the assembled PANoptosome simultaneously regulates the execution proteins involved in apoptosis, pyroptosis and necroptosis.^[^ ^10^ ^]^ Cleaved caspase‐1 (Cle‐CASP1), which is activated by the PANoptosome, cleaves gasdermin D (GSDMD) to release active GSDMD‐N while also promoting the maturation of interleukin‐18 (IL18) and interleukin‐1*β* (IL1*β*); with the help of the PANoptosome, cleaved caspase‐3 (Cle‐CASP3) is transformed from precursor CASP3; Mixed‐lineage kinase domain‐like pseudokinase (MLKL) is phosphorylated by receptor‐interacting serine/threonine kinase 3 (RIPK3), which is activated by the PANoptosome.^[^ ^11^ ^]^ These findings led us to hypothesize that by regulating the PANoptosome, we might be able to simultaneously inhibit pyroptosis, apoptosis and necroptosis, thereby preserving neurons to a greater extent. Currently, the occurrence and regulatory mechanism of PANoptosis after SCI have not been elucidated and require further research.

As important RNA posttranscriptional modification factors, RNA‐binding proteins (RBPs) are widely involved in activities such as gene expression, RNA splicing, and RNA degradation.^[^ ^12^ ^]^ Y‐box‐binding protein 1 (YBX1) is a multifunctional protein in the RBP family that contains an evolutionarily conserved cold shock domain.^[^ ^13^ ^]^ YBX1 binds and stabilizes gene transcripts, thereby mediating their upregulation.^[^ ^14^ ^]^ Moreover, YBX1 is involved in many key biological processes, including cell proliferation, differentiation, senescence and programmed cell death.^[^ ^13^ ^]^ However, research on the role of YBX1 in SCI and its regulatory mechanisms has not been conducted. Our preliminary omics data revealed that YBX1 is highly expressed in neurons after SCI. Thus, the role of YBX1 and its association with PANoptosis in SCI deserve further investigation, and YBX1 may serve as a target for treatment.

Ubiquitination is a crucial posttranslational modification that regulates the degradation of intracellular proteins and is widely involved in various important biological processes.^[^ ^15^ ^]^ Ubiquitination is reversible and is determined by the cooperation between ubiquitinases and deubiquitinases. RING domain‐containing E3 ubiquitin ligases belong to the tripartite motif (TRIM)‐containing protein family.^[^ ^16^ ^]^ Although only a few members have been investigated, novel insights into the role of the TRIM family in ubiquitination have been provided. This family of proteins has been found to be involved in the regulation of tumor immunity, stress granule homeostasis, viral infection, and nonalcoholic steatohepatitis.^[^ ^17^ ^]^ However, whether ubiquitination mediated by the TRIM family is associated with SCI remains unknown. In the present study, we performed a series of experiments to explore the important molecular factors that control the progression of pathology after SCI. Specifically, we aimed to confirm 1) whether YBX1 is an important factor involved in pathological progression after SCI; 2) whether a relationship exists between YBX1 and neuronal PANoptosis and the potential mechanism underlying this relationship; and 3) whether TRIM family members are involved in regulating the stability of YBX1 in neurons.

## 2 Results

### 2.1 Proteomic Data Reveal an Association Between SCI and YBX1‐Mediated RNA Stability

**Figure 1:**
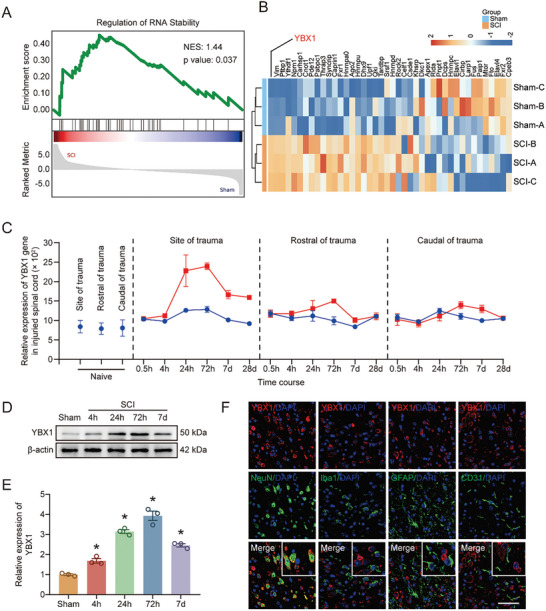
Proteomic data reveal an association between SCI and YBX1‐mediated RNA stability. A) GSEA for Regulation of RNA Stability in SCI versus sham (n = 3). B) The heatmap showed all genes enriched by GSEA (n = 3). C) The transcriptome data from GDS2159/1418624_at was downloaded to analyze the expression profile of YBX1. D,E) Western blot and quantification of the temporal expression profile of YBX1 in spinal cord tissue (n = 3). F) Co‐localization analysis of YBX1 with neurons (NeuN^+^), astrocytes (GFAP^+^), microglia (Iba1^+^) and endothelial cells (CD31^+^) after SCI (Scale bar: 50 µm). The data are presented as the means ± SEMs; *p < 0.05. Significance for Figure 1A was calculated using a two‐tailed, unpaired t test. Significance for Figure 1E was calculated using two‐way ANOVA combined with Tukey's multiple comparison test.

We performed a proteomic analysis to investigate the pathological changes that occur after SCI. GSEA revealed that mRNA stability changed significantly in the injured spinal cord (**Figure** **1**A). Furthermore, among the proteins related to mRNA stability, YBX1 had the highest enrichment score (Figure 1B). We then analyzed a GEO dataset and performed immunohistochemistry and western blot experiments to obtain a deeper understanding of the spatiotemporal distribution and expression of YBX1 after SCI. Gene expression data from the GDS2159/1418624 datasets (https://www.ncbi.nlm.nih.gov/geo/tools/profileGraph.cgi?ID=GDS2159:1418624_at) revealed that YBX1 was differentially expressed in the injured area, and YBX1 gene expression began to increase 24 h after injury and peaked at 72 h (Figure 1C). Consistently, the western blot results also revealed that YBX1 protein expression reached its highest level at 72 h after injury (Figure 1D,E). In addition, through immunohistochemistry, we determined that YBX1 was highly expressed in neurons after injury (Figure 1F). These results suggest that YBX1 may play an important regulatory role in neuronal survival after SCI.

### 2.2 YBX1 Knockdown Facilitates Functional Recovery After SCI

**Figure 2:**
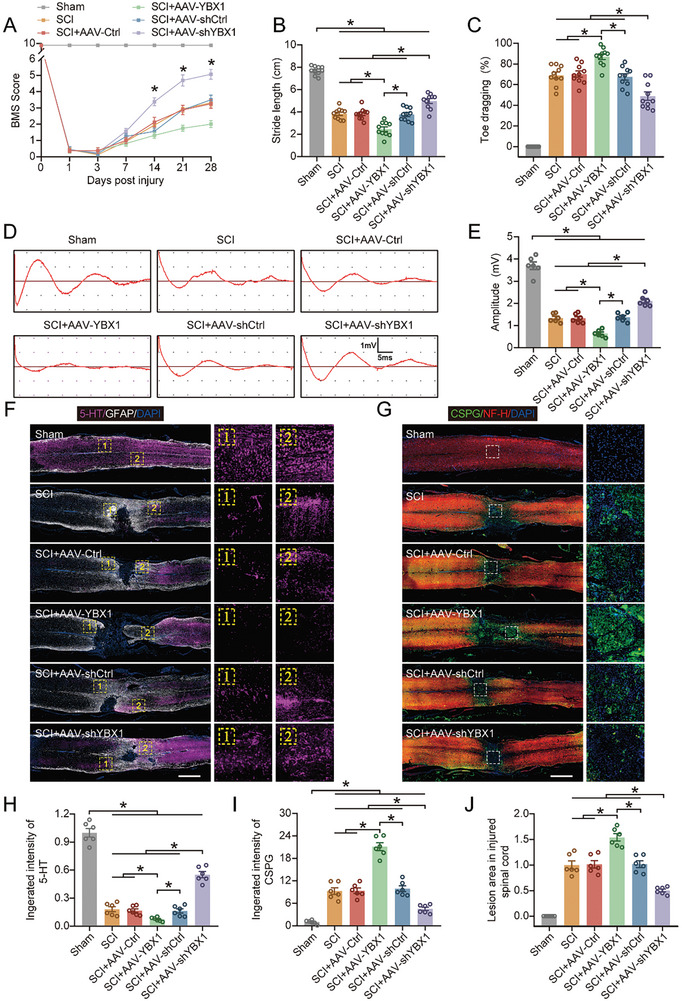
YBX1 knockdown facilitates functional recovery after SCI. A) BMS scores were recorded and analyzed at the indicated time points after SCI (n = 10). B,C) Quantification of stride width and toe drag in footprint analysis on day 28 after SCI (n = 10). D,E) Motor evoked potentials were recorded in mice on day 28 after SCI using electromyography and their amplitudes were quantified (n = 6). F,H) Immunohistochemical staining and quantification results of 5‐HT and GFAP in sagittal sections of thoracic spinal cord at 28 days (n = 6; scale bar: 1000 µm). Enlarged images of the boxed areas were shown on the right. G,I) Immunohistochemical staining and quantification results of CSPG and NF‐H in sagittal sections of thoracic spinal cord at 28 days (n = 6). Enlarged images of the boxed areas were shown on the right. J) Quantification of lesion area in sagittal sections of thoracic spinal cord at 28 days (n = 6; scale bar: 1000 µm). The data are presented as the means ± SEMs; *p < 0.05. Significance was calculated using two‐way ANOVA combined with Tukey's multiple comparison test.

We used AAV to overexpress or knock down YBX1 in neurons and explore its role after SCI. The transfection efficiency of the AAVs was verified by western blot and qPCR, and the results revealed that both knockdown and overexpression were successful (Figure S1A–C, Supporting Information). We then established a SCI model in all the mice and assessed their neurological function. The functional behavioral analysis, which included BMS scores and the footprint analysis, demonstrated significantly improved motor function recovery in YBX1–knockdown mice (**Figure** **2**A–C; Figure S2A, Supporting Information). The electromyography analysis revealed that YBX1–knockdown mice presented greater motor‐evoked potential amplitudes (Figure 2D,E). We also used immunohistochemistry to observe the pathophysiological changes in the injured area. We found that in YBX1–knockdown mice, the number of descending 5‐HT‐positive axon fibers was significantly greater after SCI, whereas the CSPG‐positive area was significantly reduced (Figure 2F–I). The histopathological analysis revealed that YBX1 knockdown significantly reduced the lesion area (Figure 2J). Furthermore, the number of surviving neurons was significantly greater in the YBX1‐knockdown mice than in the YBX1‐overexpressing mice (Figure S3A,B, Supporting Information). In summary, the above results suggest that YBX1 knockdown is beneficial for functional repair after SCI.

### 2.3 YBX1 Regulates Neuronal PANoptosis After SCI by Regulating the Stability of *Zbp1*

**Figure 3:**
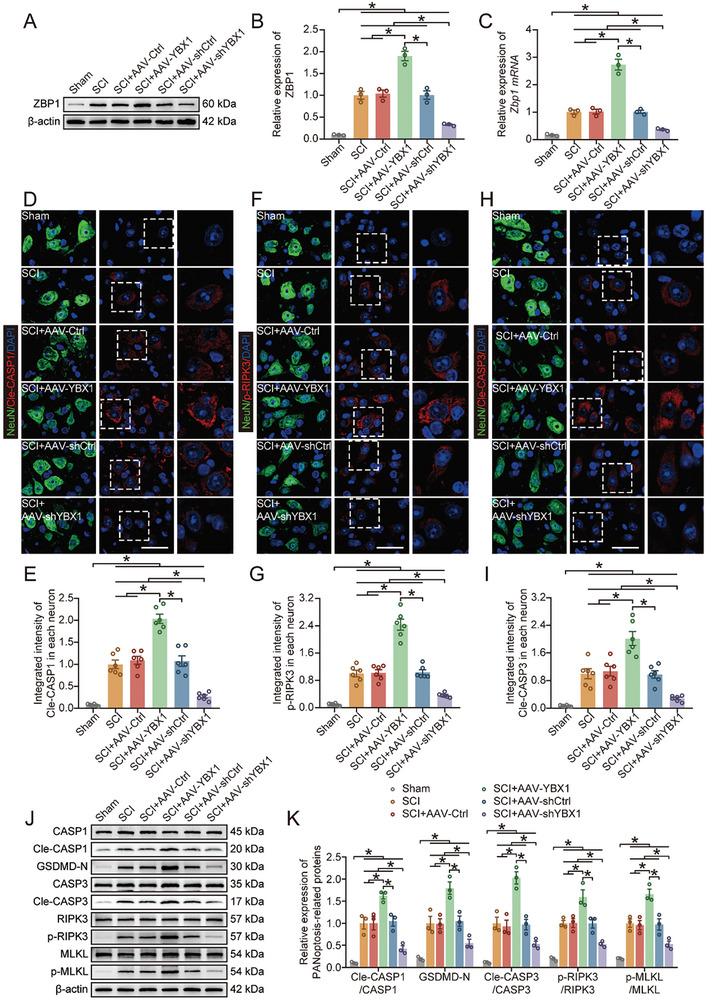
YBX1 regulates PANoptosis after SCI by regulating the stability of *Zbp1*. A,B) Western blot and quantification of ZBP1 expression in spinal cord tissue on day 3 after SCI (n = 3). C) qPCR analysis of *Zbp1* abundance in spinal cord tissue on day 3 after SCI (n = 3). D–I) Immunohistochemical staining and quantification results of Cle‐CASP1, p‐RIPK3 and Cle‐CASP3 in neurons in coronal sections of thoracic spinal cord on day 3 after SCI (n = 6; scale bar: 30 µm). J,K) Western blot and quantification of PANoptosis‐related protein expression in spinal cord tissue on day 3 after SCI (n = 3). The data are presented as the means ± SEMs; *p < 0.05. Significance was calculated using two‐way ANOVA combined with Tukey's multiple comparison test.

YBX1 is an important intracellular RBP, and thus we hypothesized that YBX1 may influence the prognosis of mice with SCI by affecting related RNAs. We performed RIP experiments followed by RNA sequencing to determine the RNA bound by YBX1. The integrative genomics viewer revealed greater enrichment of *Zbp1* in the YBX1‐RIP group than in the IgG group (Figure S4A, Supporting Information). This result was also supported by the results of the RIP‐PCR experiments (Figure S4B, Supporting Information). We also performed experiments to determine whether regulating YBX1 expression in vivo affects the abundance of *Zbp1* mRNA and protein expression of ZBP1. Western blot and qPCR experiments revealed that the abundance of *Zbp1* mRNA and protein expression of ZBP1were correlated with changes in YBX1 expression (**Figure** **3**A–C). These results indicate that YBX1 promotes ZBP1 protein expression by increasing the stability of *Zbp1*.

ZBP1 has been shown to be involved in PANoptosis, a new form of cell death established based on extensive crosstalk between pyroptosis, apoptosis, and necroptosis.^[^ ^19^ ^]^ Interestingly, our previous studies revealed the activation of pyroptosis, apoptosis and necroptosis after SCI;^[^ ^18^, ^20^ ^]^ therefore, we hypothesized that PANoptosis occurs in SCI and that ZBP1 may mediate this process. The immunohistochemistry results suggested that decreased expression of ZBP1 alleviated the activation of Cle‐CASP1, Cle‐CASP3 and p‐RIPK3 in neurons, whereas increased expression of ZBP1 promoted the activation of these executive molecules (Figure 3D–I). Western blot analysis revealed that when ZBP1 expression was inhibited, the levels of Cle‐CASP1, GSDMD‐N, Cle‐CASP3, p‐RIPK3 and p‐MLKL were significantly reduced (Figure 3J,K). Moreover, ELISAs were performed to measure the levels of PANoptosis‐related inflammatory factors, and the results showed that the levels of IL‐18 and IL‐1*β* were significantly reduced after ZBP1 expression was inhibited (Figure S5A,B, Supporting Information).

We then designed rescue experiments to further determine the relationship between YBX1 and ZBP1‐mediated PANoptosis. We injected AAVs designed to overexpress YBX1 or knockdown ZBP1 into the target spinal cord segment via in situ injection. The western blot and qPCR results revealed that both AAV‐YBX1 and AAV‐shZBP1 were successfully transfected (Figure S6A–E, Supporting Information). We also found that transfection with AAV‐YBX1 partially restored the expression of ZBP1, which was suppressed after transfection with AAV‐shZBP1, but transfection with AAV‐shZBP1 had no effect on YBX1 expression (Figure S6A–E, Supporting Information). In addition, the western blot results showed that ZBP1 knockdown effectively inhibited the increases in the levels of Cle‐CASP1, GSDMD‐N, Cle‐CASP3, p‐RIPK3 and p‐MLKL caused by YBX1 overexpression (Figure S7A,B, Supporting Information). The ELISA results were consistent with those described above. ZBP1 knockdown effectively alleviated the increases in IL‐18 and IL‐1*β* levels caused by YBX1 overexpression (Figure S7C,D, Supporting Information). Together, these results indicate that ZBP1‐mediated PANoptosis occurs in neurons after SCI and is modulated by YBX1.

### 2.4 TRIM56 Interacts with YBX1

**Figure 4:**
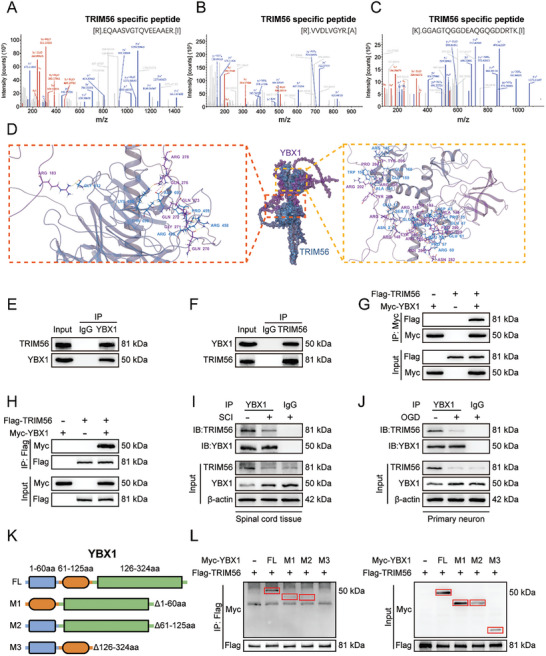
TRIM56 interacts with YBX1. A–C) The TRIM56‐specific peptide in the IP product of YBX1 was discovered through IP/MS analysis. D) Molecular docking analysis of TRIM56 and YBX1. E,F) Co‐IP analysis of the interaction between YBX1 and TRIM56 in neurons was performed with anti‐YBX1 or anti‐TRIM56 primary antibodies (n = 3). G,H) HEK293T cells were co‐transfected with Flag‐TRIM56 and Myc‐YBX1, and the interaction between YBX1 and TRIM56 was analyzed by Co‐IP using anti‐Flag or anti‐Myc primary antibodies (n = 3). I) Co‐IP of TRIM56 and YBX1 in the spinal cord tissue of mice undergoing SCI surgery or sham surgery (n = 3). J) Co‐IP of TRIM56 and YBX1 in primary neurons with or without OGD treatment (n = 3). K) Schematic illustration of YBX1 and its truncated mutants. L) Myc‐tagged YBX1 or its truncated mutants was co‐transfected with Flag‐TRIM56 into HEK293T cells. Cell lysates were Co‐IP with anti‐Myc antibody, followed by Western blot detection (n = 3). The data are presented as the means ± SEMs; *p < 0.05. Significance was calculated using two‐way ANOVA combined with Tukey's multiple comparison test.

Next, we aimed to determine the underlying mechanisms regulating YBX1 expression. The ubiquitin‐proteasome degradation pathway and the autophagy‐lysosomal pathway are two important pathways for intracellular protein degradation. 3MA is a widely used inhibitor of autophagy that inhibits class III PI3K.^[^ ^21^ ^]^ CQ disrupts lysosomal function by increasing the pH of acidic endosomes/lysosomes.^[^ ^22^ ^]^ MG132 is a widely used ubiquitin‐proteasome degradation pathway inhibitor.^[^ ^15a^ ^]^ We treated primary neurons (Figure S8A, Supporting Information) with the proteasome inhibitor MG132, the autophagy inhibitor 3MA and the lysosome inhibitor CQ. The western blot analysis revealed increased YBX1 expression after MG132 treatment, suggesting that the ubiquitin‐proteasome degradation pathway may be the main regulatory mechanism of YBX1 in neurons (Figure S9A, Supporting Information). Next, we performed IP/MS and molecular docking analyses to identify molecules that interact with YBX1. Interestingly, the results suggested that the E3 ubiquitin ligase TRIM56 strongly binds to YBX1 (**Figure** **4**A–D). Co‐IP was also performed to verify the above results. YBX1 precipitated TRIM56 but not IgG in neurons (Figure 4E). Reverse Co‐IP confirmed that TRIM56 effectively bound and precipitated YBX1 in neurons (Figure 4F). We then cotransfected TRIM56‐Flag and YBX1‐Myc into HEK293T cells and performed Co‐IP using antibodies against the corresponding tags. Consistently, Flag‐tagged TRIM56 and Myc‐tagged YBX1 strongly bound to each other in HEK293T cells (Figure 4G,H).

Additionally, we observed that the expression of TRIM56 and its binding to YBX1 were decreased in both SCI model mice and OGD‐treated neurons (Figure 4I,J). Finally, we constructed a series of truncated Myc‐tagged YBX1 plasmids and cotransfected HEK293T cells with a Flag‐tagged full‐length TRIM56 plasmid to determine to which region of YBX1 TRIM56 specifically binds. As shown by the Co‐IP results, TRIM56 only bound to the M3 (Δ126‐324) fragment of YBX1 (Figure 4K,L). These results indicate a direct interaction between TRIM56 and YBX1.

### 2.5 TRIM56 Promotes the Ubiquitination and Degradation of YBX1

**Figure 5:**
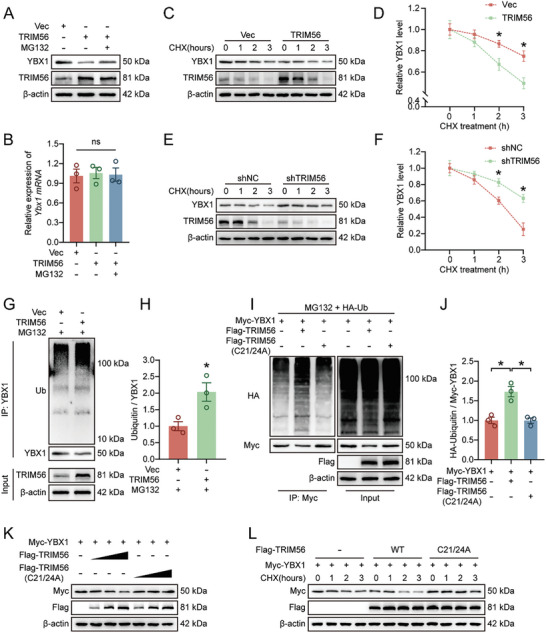
TRIM56 promotes ubiquitination and degradation of YBX1. A) HEK293T cells were transfected with TRIM56 plasmid to overexpress TRIM56. The expression of YBX1 and TRIM56 with or without MG132 treatment was analyzed by Western blot (n = 3). B) qPCR analysis of YBX1 in HEK293T cells in indicated groups (n = 3). C,D) Western blot and quantification of YBX1 in CHX‐treated (100 µg mL^−1^) Control or TRIM56‐overexpressing HEK293T cells (n = 3). E,F) Western blot and quantification of YBX1 in CHX‐treated (100 µg mL^−1^) Control or TRIM56 knockdown HEK293T cells (n = 3). G,H) Western blot and quantification of ubiquitination of YBX1 in HEK293T cells overexpressing TRIM56 (n = 3). Cells were pretreated with 10 µm MG132 for 6 h before harvest. I,J) HA‐Ub and Myc‐YBX1 were co‐transfected with Flag‐TRIM56 or Flag‐TRIM56 (C21/24A) in HEK293T cells. YBX1 ubiquitination in cells was analyzed and quantified by Western blot (n = 3). Cells were pretreated with 10 µm MG132 for 6 h before harvest. K) Myc‐YBX1 was co‐transfected with different concentrations of Flag‐TRIM56 or Flag‐TRIM56 (C21/24A) plasmid into HEK293T cells and lysates from these cells were analyzed by western blot (n = 3). L) Myc‐YBX1 was co‐transfected with Flag‐TRIM56 or Flag‐TRIM56 (C21/24A) plasmid into HEK293T cells. Western blot analysis was performed using corresponding antibodies after CHX (100 µg mL^−1^) treatment (n = 3). The data are presented as the means ± SEMs; *p < 0.05. Significance for Figure 5G was calculated using a two‐tailed, unpaired t test. Significance for Figure 5B,D,F,J was calculated using two‐way ANOVA combined with Tukey's multiple comparison test.

We continued to conduct relevant experiments to further clarify whether regulating TRIM56 affects the ubiquitination and degradation of YBX1. We found that the addition of MG132 abrogated the decrease in YBX1 expression caused by the overexpression of TRIM56, whereas these treatments had no effect on the *Ybx1* mRNA level (**Figure** **5**A,B). CHX was used to inhibit intracellular protein synthesis, and in this case, the effects of TRIM56 overexpression or knockdown on the stability of the endogenous YBX1 protein were evaluated. We found that the overexpression of TRIM56 significantly shortened the half‐life of YBX1, whereas silencing the expression of TRIM56 significantly prolonged the half‐life of YBX1 (Figure 5C–F). Consistently, the ubiquitination level of YBX1 was significantly increased after TRIM56 overexpression, whereas the expression of YBX1 was significantly decreased (Figure 5G,H). In accordance with previous studies, an E3 ubiquitin ligase activity‐defective mutant was constructed in this study.^[^ ^16^ ^]^ We cotransfected HEK293T cells with YBX1‐Myc, Ub‐HA and TRIM56‐Flag (WT or C21/24A mutant) to further clarify the regulatory effect of TRIM56 on YBX1 ubiquitination. As shown in Figure 5I, overexpression of TRIM56 significantly promoted the ubiquitination and degradation of YBX1, but the transfection of C21/24A mutant TRIM56 did not have this effect (Figure 5J). Similarly, TRIM56 overexpression decreased YBX1 expression, whereas overexpression of the C21/24A mutant had no such effect (Figure 5K). In addition, the shortening of the YBX1 half‐life caused by the overexpression of TRIM56 did not occur when the C21/24A mutant was overexpressed (Figure 5L). Taken together, these results indicate that TRIM56 directly ubiquitinates YBX1 to promote its proteasomal degradation.

### 2.6 Knocking Down TRIM56 Exacerbates Neuronal PANoptosis and Limits Functional Recovery After SCI by Promoting YBX1 Expression

We injected AAVs for YBX1 or TRIM56 knockdown into the target spinal cord segment via in situ injection to verify that TRIM56 promotes functional recovery after SCI by regulating YBX1‐mediated PANoptosis. The transfection efficiency of AAV‐shYBX1 was verified previously (Figure S1A–C, Supporting Information), and AAV‐shTRIM56 was successfully transfected in this part (Figure S10A–E, Supporting Information). We found that the transfection of AAV‐shTRIM56 partially restored the expression of YBX1, which was inhibited after AAV‐shYBX1 transfection, but AAV‐shYBX1 transfection had no effect on the expression of TRIM56 (Figure S10A–E, Supporting Information).

**Figure 6:**
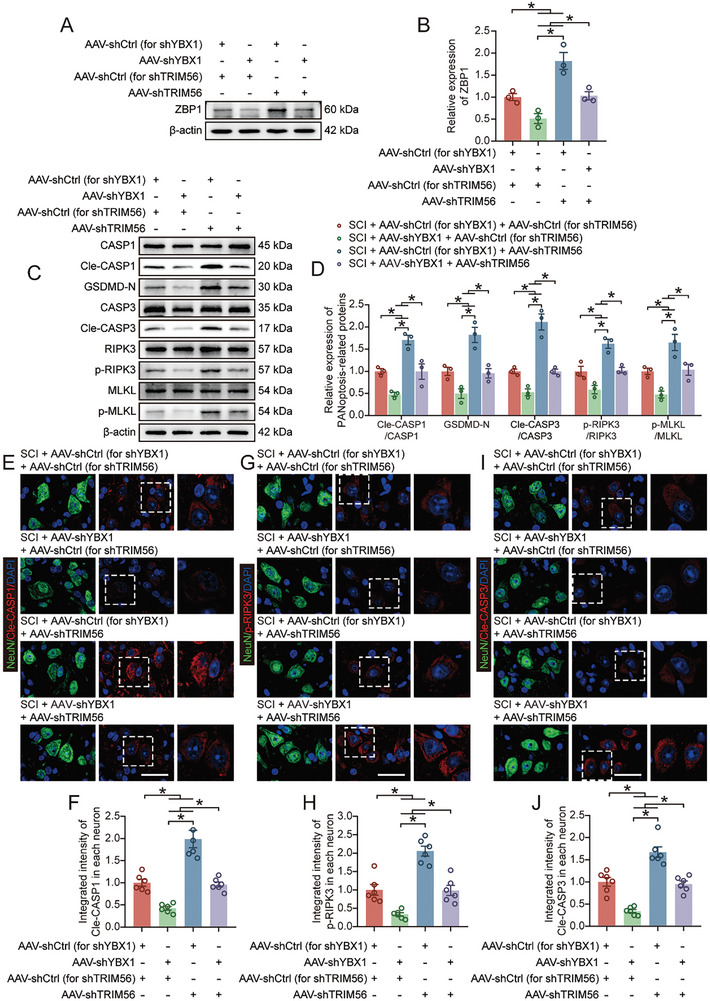
Knocking down TRIM56 exacerbates PANoptosis by promoting YBX1 expression. A,B) Western blot and quantification of ZBP1 expression in spinal cord tissue on day 3 after SCI (n = 3). C,D) Western blot and quantification of PANoptosis‐related protein expression in spinal cord tissue on day 3 after SCI (n = 3). E–J) Immunohistochemical staining and quantification results of Cle‐CASP1, p‐RIPK3 and Cle‐CASP3 in neurons in coronal sections of thoracic spinal cord on day 3 after SCI (n = 6; scale bar: 30 µm). The data are presented as the means ± SEMs; *p < 0.05. Significance was calculated using two‐way ANOVA combined with Tukey's multiple comparison test.

**Figure 7:**
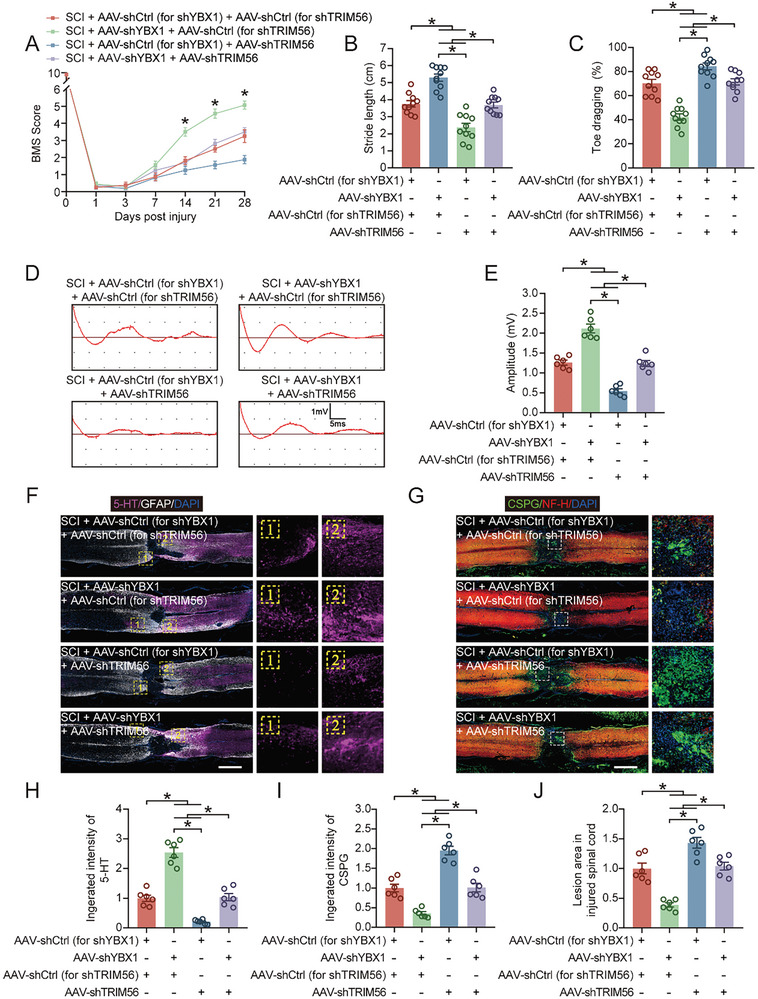
Knocking down TRIM56 limits functional recovery after SCI. A) BMS scores were recorded and analyzed at the indicated time points after SCI (n = 10). B,C) Quantification of stride width and toe drag in footprint analysis on day 28 after SCI (n = 10). D,E) Motor evoked potentials were recorded in mice on day 28 after SCI using electromyography and their amplitudes were quantified (n = 6). F,H) Immunohistochemical staining and quantification results of 5‐HT and GFAP in sagittal sections of thoracic spinal cord at 28 days (n = 6; scale bar: 1000 µm). Enlarged images of the boxed areas were shown on the right. G,I) Immunohistochemical staining and quantification results of CSPG and NF‐H in sagittal sections of thoracic spinal cord at 28 days (n = 6; scale bar: 1000 µm). Enlarged images of the boxed areas were shown on the right. J) Quantification of lesion area in sagittal sections of thoracic spinal cord at 28 days (n = 6). The data are presented as the means ± SEMs; *p < 0.05. Significance was calculated using two‐way ANOVA combined with Tukey's multiple comparison test.

Next, we examined the level of PANoptosis in the spinal cord tissue. The restoration of YBX1 expression by the inhibition of TRIM56 expression also restored *Zbp1* mRNA and protein expression of ZBP1 (**Figure** **6**A,B; Figure S11A, Supporting Information). The levels of PANoptosis‐related proteins and inflammatory factors were significantly downregulated after AAV‐shYBX1 treatment, while AAV‐shTRIM56 treatment partially restored their levels (Figure 6C,D; Figure S12A,B, Supporting Information). Moreover, the immunohistochemical analysis revealed that AAV‐shTRIM56 treatment partially abrogated the pro‐neuronal survival effect of AVV‐shYBX1 treatment and aggravated neuronal PANoptosis (Figure 6E–J). Neurological function‐related tests were conducted to further understand the role of TRIM56 in functional recovery after SCI. The functional behavioral analysis (BMS scores and footprint analysis) and electromyography analysis revealed that cotreatment with AAV‐shYBX1 and AAV‐shTRIM56 partially reduced functional recovery compared with that in the AAV‐shYBX1 treatment group (**Figure** **7**A–E; Figure S13A, Supporting Information). The immunohistochemical analysis of spinal cord tissue suggested that cotreatment with AAV‐shYBX1 and AAV‐shTRIM56 counteracted the effect of AAV‐shYBX1 alone on promoting neurological function (Figure 7F–I). Furthermore, compared with AAV‐shYBX1 treatment alone, AAV‐shYBX1 and AAV‐shTRIM56 cotreatment expanded the lesion area and significantly reduced the number of surviving neurons (Figure 7J; Figure S14A,B, Supporting Information). Collectively, these results indicate that TRIM56 limits ZBP1‐mediated neuronal PANoptosis by regulating the degradation of YBX1, thereby affecting the prognosis of mice with SCI.

## 3 Discussion

The treatment of SCI has always been a major medical challenge. Despite many attempts, the complex spatiotemporal pathophysiological changes that occur after SCI have prevented the development and large‐scale clinical translation of effective therapeutic agents. Efforts to understand the complex pathological processes that occur after SCI are urgently needed. In the present study, for the first time, we investigated the role of YBX1, an RBP, in traumatic SCI. We used loss‐of‐function and gain‐of‐function methods to prove that YBX1 affects the prognosis of mice with SCI by regulating ZBP1‐mediated neuronal PANoptosis, which has not been previously reported. In addition, with the help of experiments to assess molecular interactions, we also found that TRIM56 is the main E3 ubiquitin ligase that regulates YBX1 degradation in neurons. These results suggest that the TRIM56‐YBX1‐PANoptosis axis may be a potential target for the treatment of SCI.

YBX1 is considered an oncogene in most reports. By regulating mRNA stability, YBX1 affects the proliferation, invasion and migration of cancer cells.^[^ ^23^ ^]^ YBX1 recruited by the circular RNA ACTN4 promotes intrahepatic cholangiocarcinoma progression by initiating FZD7 transcription.^[^ ^24^ ^]^ Additionally, YBX1 promotes the development of esophageal squamous cell carcinoma by stabilizing the SMOX mRNA.^[^ ^25^ ^]^ In recent years, the role of YBX1 in noncancer diseases has gradually emerged, and studies of the role of YBX1 in cardiac function and adipocyte proliferation have been reported.^[^ ^26^ ^]^ In mouse white fat, YBX1 overexpression enhances ULK1/ULK2‐mediated autophagy and promotes adipose tissue expansion.^[^ ^27^ ^]^ However, the role of YBX1 in traumatic SCI remains unclear. The proteomic analysis revealed significant changes in mRNA stability within the damaged spinal cord segments, and among the enriched RBPs, YBX1 had the highest score. We then further clarified the spatiotemporal expression pattern of YBX1. The GEO database analysis revealed that YBX1 expression increased in the injured area and peaked on the third day after injury, which was also supported by the results of western blot experiments. Additionally, the results of the immunohistochemical analysis suggested that YBX1 was upregulated mainly in neurons. In addition, functional recovery was significantly improved after neuronal YBX1 was knocked down. These results indicate that YBX1 is a potential target for the treatment of SCI, but the specific mechanisms involving YBX1 require further study.

Neuronal loss is considered one of the most important causes of poor functional recovery after SCI, and previous studies have shown that inhibiting programmed neuronal cell death is beneficial for neuronal preservation and functional recovery.^[^ ^6^, ^28^ ^]^ PANoptosis is a new form of cell death recently proposed based on the extensive crosstalk among apoptosis, pyroptosis and necroptosis.^[^ ^29^ ^]^ Under the stimulation of certain signals, the PANoptosome is assembled to further activate the downstream executive molecules GSDMD, MLKL and CASP3 and ultimately induces inflammatory cell death.^[^ ^7^, ^30^ ^]^ Some researchers have observed the occurrence of neuronal PANoptosis in SCI,^[^ ^31^ ^]^ but its specific molecular mechanism has not yet been elucidated. Considering that MLKL, GSDMD, and CASP3 are all regulated by the PANoptosome, inhibiting the occurrence of neuronal PANoptosis may simultaneously block the processes of apoptosis, pyroptosis and necroptosis, which may have greater significance in the promotion of neuronal survival after SCI. The PANoptosome is a multiprotein complex, and its assembly is regulated by multiple factors. Among the constituent members, ZBP1 is considered one of the important molecules driving PANoptosome assembly.^[^ ^32^ ^]^ Interestingly, using RIP‐sequencing technology, we detected strong binding between YBX1 and *Zbp1*. We then conducted further verification experiments, and the results revealed that ZBP1‐mediated PANoptosis occurred in neurons after SCI and that YBX1 increased the expression of ZBP1 by binding and stabilizing *Zbp1*. These data clarify for the first time that YBX1 regulates ZBP1‐mediated PANoptosis in SCI, providing a new perspective on the pathological mechanism of SCI.

Although we have revealed the important roles of YBX1 and ZBP1‐mediated PANoptosis in SCI, to our knowledge, YBX1 inhibitors are currently unavailable. We aimed to clarify the mechanism by which the stability of YBX1 is regulated in vivo to further understand the mechanism underlying changes in YBX1 expression and provide a basis for possible future targeted drug development. Using specific inhibitors (3MA, CQ and MG132), we found that only MG132 effectively inhibited the degradation of YBX1, suggesting that YBX1 in spinal neurons is regulated mainly by the ubiquitin‐proteasome pathway. Previous studies have shown that E3 ubiquitin ligases such as Nedd4L and PRP19 promote the ubiquitination and degradation of YBX1, whereas the deubiquitinating enzyme USP47 inhibits the ubiquitination of YBX1.^[^ ^14^, ^33^ ^]^ Here, we used IP experiments and molecular docking analysis to determine the binding affinity between TRIM56 and YBX1.

TRIM56 is a RING domain‐containing E3 ubiquitin ligase that belongs to the TRIM‐containing protein family.^[^ ^34^ ^]^ The important role of TRIM56 in some diseases has been reported. Upon viral infection, TRIM56 is an essential component of the cytoplasmic DNA‐sensing pathway that induces anti‐DNA virus innate immunity.^[^ ^35^ ^]^ In glioma, TRIM56 promotes CDC42 activation by regulating IQGAP1 ubiquitination, thereby promoting glioma cell migration.^[^ ^34^ ^]^ Additionally, overexpression of TRIM56 enhances the activation of TNF*α*‐induced NF‐κB signaling by promoting the ubiquitination of TAK1.^[^ ^36^ ^]^ At present, several members of the TRIM‐containing protein family have been proven to regulate protein ubiquitination and degradation.^[^ ^37^ ^]^ However, the role of TRIM56 in SCI and whether it regulates the expression of YBX1 remain unclear. A series of in vitro experiments on YBX1 stability confirmed that TRIM56 increases the ubiquitination of YBX1 to promote its degradation via the proteasomal pathway. We then verified in vivo whether TRIM56 modulates YBX1‐mediated neuronal PANoptosis. We observed that inhibiting TRIM56 expression increased YBX1 levels, suggesting that TRIM56 is a modulator of YBX1 degradation in vivo. In addition, the increase in neuronal PANoptosis caused by the inhibition of TRIM56 was alleviated after the inhibition of YBX1 expression, indicating that TRIM56 indeed affects neuronal survival by modulating YBX1‐mediated PANoptosis. These results indicate for the first time that TRIM56 is an alternative endogenous YBX1 modulator that promotes functional recovery after SCI by regulating YBX1‐mediated neuronal PANoptosis. However, the specific binding site and ubiquitination mode between TRIM56 and YBX1 require more refined structural biology studies.

Overall, our findings are meaningful and have clinical translational implications. In recent years, the development of in vivo gene regulation technologies such as RNA vaccines has been exciting.^[^ ^38^ ^]^ Regulating the activity of the TRIM56‐YBX1‐PANoptosis axis in vivo through technologies such as RNA vaccines may be a feasible method for clinical transformation. The preparation of relevant RNA vaccines and their functional verification in animal SCI models is worthy of further research. In addition, a recent study characterized neurons in the spinal cord that control hind limb walking and achieved significant motor function recovery in mice with spinal cord transection.^[^ ^39^ ^]^ Targeted regulation of the TRIM56‐YBX1‐PANoptosis axis in these characterized neurons to promote their survival will be a very promising strategy for SCI treatment, and the technology of targeted regulation of characterized neurons awaits follow‐up research. Although these potential treatment strategies are encouraging, several questions remain to be addressed before relevant trials can be implemented. Whether RNA vaccines have a strong ability to target specific areas of SCI requires rigorous experiments for verification. Considering that RNA vaccines may inevitably leak into other parts of the spinal cord and other tissues, the safety and possible side effects of the vaccine also need to be strictly tested and verified. In addition, characteristic neurons have only been found in mice thus far. Whether such neurons exist in the human spinal cord and how to locate and target these neurons in the human spinal cord remain to be further clarified.

Our study still has some limitations that deserve further investigation. The proinflammatory death of glial cells also plays an important role in the pathological process after SCI.^[^ ^40^ ^]^ The present study focused only on neuronal PANoptosis, and the role of PANoptosis in microglia and astrocytes needs to be further clarified. Previous studies have shown that AIM2 and NLRP12 are also important upstream factors regulating PANoptosis.^[^ ^30^, ^32^ ^]^ In this study, we verified only ZBP1‐mediated PANoptosis in neurons after SCI. Whether PANoptosis mediated by other factors also exists in SCI remains to be verified.

## 4 Conclusion

**Figure 8:**
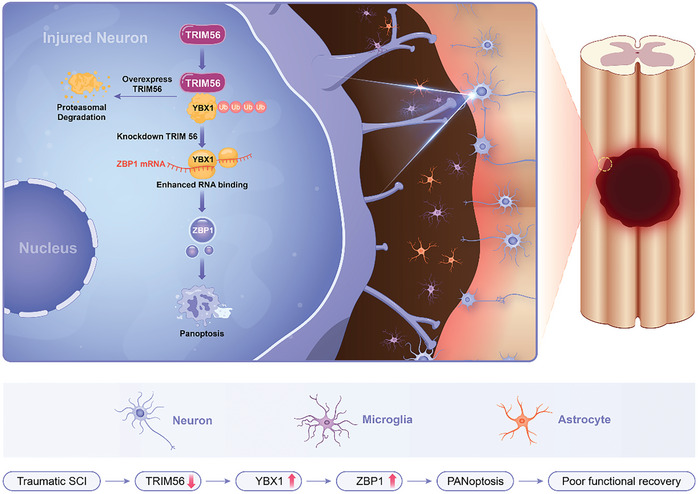
Schematic Description of the Molecular Mechanism: YBX1 stabilizes *Zbp1* mRNA, thereby promoting ZBP1 expression; TRIM56, functioning as an E3 ubiquitin ligase, modulates ZBP1‐mediated PANoptosis by regulating the ubiquitination and degradation of YBX1.

In summary, our study demonstrated that ZBP1‐mediated neuronal PANoptosis is involved in the pathological process of SCI. The interaction between TRIM56 and YBX1 is a crucial molecular event that promotes YBX1 degradation, thereby inhibiting ZBP1 expression and alleviating PANoptosis (**Figure** **8**). These findings improve our understanding of the roles of the TRIM‐containing protein family and RBPs in SCI and suggest that regulating the TRIM56‐YBX1 axis may be a promising approach for treating SCI.

## 5 Experimental Section

### Animal Procedures

All animal experiments and methods involved in the research complied with the Guide for the Care and Use of Laboratory Animals of the China National Institutes of Health and were approved by the Animal Experimental Ethical Committee of the First Affiliated Hospital College of Medicine, Zhejiang University, China (Approval No. 2023‐1411).

Healthy adult C57BL/6J mice were obtained from the Laboratory Animal Center of the First Affiliated Hospital College of Medicine, Zhejiang University, China. AAV9 harboring YBX1 shRNA (AAV9‐hSyn‐sh‐YBX1), AAV9 harboring TRIM56 shRNA (AAV9‐hSyn‐sh‐TRIM56), AAV9 harboring Z‐DNA binding protein 1 (ZBP1) shRNA (AAV9‐hSyn‐sh‐ZBP1), YBX1 overexpression vector (AAV9‐hSyn‐YBX1) and their control vector (AAV9‐hSyn‐Scrambled) were used to infect the spinal cords of the mice via in situ injection. After 3 weeks, the mice underwent spinal cord contusion surgery. At different time points after surgery, the behavioral data of the mice were recorded and analyzed, and tissue samples were collected for further research and analysis.

### Proteomic Analysis and Data Processing

Frozen samples (≈100 mg) were ground, homogenized, washed, resuspended as a lysate and reprecipitated to obtain total extractable protein. The total protein concentrations were quantified via a bicinchoninic acid assay. Based on the measured protein concentrations, a certain amount of protein was removed from each sample and diluted to the same volume and concentration. According to the amount of protein, enzymatic buffer was added and the mixture was digested at 37 °C for 12 h. After enzymatic hydrolysis, the samples were lyophilized for subsequent use.

Proteomic data were analyzed by Shanghai Luming Biological Technology Co., Ltd. (Shanghai, China). All analyses were performed with a Tims TOF Pro mass spectrometer (Thermo, Waltham, MA) equipped with an Easyspray source (Thermo, Waltham, MA). MS/MS spectra were searched using MaxQuant against the UniProt *Mus musculus* database. The specific parameters of the database search used in this study were as follows: Fixed modifications: Carbamidomethyl (C); Variable modification: Oxidation (M) and Acetyl (Protein N‐term); enzyme: trypsin; First search peptide tolerance: 20 ppm; Main search peptide tolerance: 10 ppm; Max Missed: 2. A total of 4648 proteins were identified in this analysis. The filtering conditions for differences in this project were a fold change (≥2 or ≤0.5) and a *p* value < 0.05. Compared with those in the sham group, 244 proteins were upregulated and 204 proteins were downregulated in the SCI group. The relevant datasets were then downloaded from the Molecular Signatures Database for gene set enrichment analysis (GSEA).

### Western Blot Analysis

Tissues and cells were homogenized in RIPA lysis buffer (Beyotime, Shanghai, China) supplemented with a protease inhibitor cocktail (Proteintech, Rosemont, IL) and a phosphatase inhibitor cocktail (Beyotime, Shanghai, China). The protein concentration in each sample was determined using a BCA protein assay kit (Epizyme Biotech, Shanghai, China). Approximately 20 µg of protein was subjected to electrophoresis on SDS‒PAGE gels, and the electrophoretically separated proteins were transferred to PVDF membranes (Millipore, St. Louis, MO) for the antibody binding and exposure. The antibodies used for the western blot analysis included anti‐YBX1 (1:1000, Sigma–Aldrich), anti‐ZBP1 (1:1000, Adipogen Life Sciences), anti‐CASP1 (1:1000, Adipogen Life Sciences), anti‐GSDMD‐N (1:1000, Cell Signaling Technology), anti‐CASP3 (1:1000, Cell Signaling Technology), anti‐RIPK3 (1:1000, Cell Signaling Technology), anti‐p‐RIPK3 (1:1000, Cell Signaling Technology), anti‐MLKL (1:1000, Cell Signaling Technology), anti‐p‐MLKL (1:1000, Abcam), anti‐TRIM56 (1:1000, Abcam), anti‐Flag‐Tag (1:4000, ABclonal), anti‐Myc‐Tag (1:10000, ABclonal), anti‐HA‐Tag (1:5000, ABclonal) and anti‐*β*‐actin (1:5000, Sigma–Aldrich) antibodies.

### Immunohistochemistry

After deparaffinization and hydration, the tissue sections were placed in sodium citrate buffer (0.01 m) and heated in a microwave oven to achieve antigen retrieval. The tissue sections were covered with 10% goat serum and incubated in a 37 °C incubator for 30 min to avoid antibody binding to nonspecific antigens. Then, the tissue sections were covered with a primary antibody solution diluted in 10% goat serum and incubated overnight at 4 °C. The next day, the remaining primary antibodies were washed and sections were incubated with the corresponding fluorescent dye‐conjugated secondary antibodies for 1 h at 37 °C in the dark. Finally, the remaining secondary antibodies were removed, and the fluorescence signal was detected after the sections were mounted with mounting medium. The primary antibodies used for immunohistochemistry included YBX1 (1:200, Sigma–Aldrich), NeuN (1:200, Abcam), Iba1 (1:2000, Abcam), CD31 (1:300, R&D Systems), GFAP (1:100, Santa Cruz Biotechnology), Cle‐CASP3 (1:200, Cell Signaling Technology), p‐RIPK3 (1:200, Cell Signaling Technology), Cle‐CASP1 (1:500, Adipogen Life Sciences), CSPG (1:200, Sigma–Aldrich), neurofilament H (NF‐H, 1:200, ABclonal) and 5‐hydroxytryptamine (5‐HT, 1:200, ImmunoStar) antibodies.

### Functional Behavioral Analysis

Before the behavioral test, all the mice were placed in the testing environment and allowed to adapt to the test environment for 1 h. The behavioral tests included in this experiment were Basso Mouse Scale (BMS) scores, a footprint analysis and electromyography detection.

The BMS scores were recorded from the first day after surgery and used to evaluate neurological recovery after SCI. 1, 3, 7, 14, 21, and 28 days after surgery were selected as the recording time points. The footprint analysis uses white paper to record the footprint patterns of SCI model mice to analyze motor functions. Briefly, the front paws of the mice were coated with blue dye and their back paws were coated with red dye; then, the mice encouraged to move in a straight line on white paper. After the dye on the white paper had dried, a ruler was used to measure the relevant data, and a statistical analysis of the stride length and toe dragging was performed.

On day 28 after SCI, neurological repair was assessed by recording motor‐evoked potentials from the hind limbs of the mice. The rostral end of the spinal cord was surgically exposed, and stimulating electrodes were placed there, while recording electrodes were placed at the flexor biceps femoris muscle. Additionally, a reference electrode was placed at the tendon of the distal hindlimb muscle, and a ground electrode was placed subcutaneously.

### RNA Immunoprecipitation (RIP) Sequencing

The RIP experiment was performed according to the steps in the instruction manual of the RIP kit (Sigma–Aldrich, Missouri, USA). Briefly, polysome lysis buffer, protease inhibitor and RNase inhibitor were added to the collected cell pellets, which were placed on ice for lysis. The lysate was subjected to DNA removal, and the supernatant was collected by centrifugation and placed in a new RNase‐free centrifuge tube. The target primary antibody was added to the supernatant to perform the antigen‐antibody binding reaction, and then balanced Protein A/G beads were added to capture the antigen‐antibody complex. Finally, the Protein A/G beads were collected and washed, and RNA was extracted using the TRIzol method. For sequencing, 20 µL of each RNA sample was subjected to high‐throughput sequencing to detect the RNA type. Sequencing was performed at OE Biotech.

### Quantitative PCR (qPCR) Analysis

Total RNA was extracted from cells and spinal cord tissues using TRIzol (Takara, Shiga, Japan). One microgram of RNA was used for reverse transcription to generate cDNA. The RNA levels of genes of interest were detected by qPCR as described previously.^[^ ^18^ ^]^ The relative expression of each gene was calculated using the 2^−ΔΔCT^ method and normalized to the *β‐actin* level. The sequences of primers used in this study are listed in Table S1 (Supporting Information).

### Enzyme‐Linked Immunosorbent Assay (ELISA)

The protein levels of IL‐18 (Servicebio, Wuhan, China) and IL‐1*β* (Servicebio, Wuhan, China) in the spinal cord tissue were detected using ELISA kits according to the manufacturer's protocol. The optical density of each sample was measured at a wavelength of 450 nm using a microplate reader. The expression level of the target protein in the sample was calculated from the standard curve.

### Cell Culture and Primary Neuron Isolation

HEK293T cells (Cat#: FH0244) were purchased from the National Collection of Authenticated Cell Cultures (Shanghai, China). HEK293T cells were cultured in Dulbecco's modified Eagle's medium (DMEM; Gibco, Waltham, MA). Ten percent fetal bovine serum (FBS; SERANA, Brandenburg, Germany) was added to the medium as a supplement. Primary neurons were isolated via trypsin digestion. Briefly, minced neural tissue was digested with 0.05% trypsin (Gibco, Waltham, MA) containing 20 U mL^−1^ DNase (Beyotime, Shanghai, China). The digestion process was performed in a 37 °C incubator, with mixing every 4 min for a total digestion of 12 min. An equal amount of DMEM containing 10% FBS was added to stop the digestion, and the supernatant was carefully pipetted into a 15 mL centrifuge tube. The cells in the supernatant were counted and seeded into poly‐L‐lysine (Beyotime, Shanghai, China)‐coated cell culture dishes. After 24 h, the culture medium was replaced with neurobasal medium (Gibco, Waltham, MA) supplemented with 2% B27 (Gibco, Waltham, MA) and 1% GlutaMAX‐I (Gibco, Waltham, MA). Half of the culture medium was changed every 2–3 days throughout the culture period. MG132, chloroquine (CQ; MedChemExpress, Monmouth Junction, NJ), 3‐methyladenine (3MA; MedChemExpress, Monmouth Junction, NJ) and cycloheximide (CHX; MedChemExpress, Monmouth Junction, NJ) were used in specific experiments.

### Plasmid Construction and Transfection

TRIM56‐Flag, TRIM56‐C21/24A‐Flag, and shTRIM56 plasmids were constructed by Genechem (Shanghai, China). Ubiquitin (Ub)‐HA, YBX1‐Myc, YBX1‐(Δ1‐60aa)‐Myc, YBX1‐(Δ61‐125aa)‐Myc, and YBX1‐(Δ126‐324aa)‐Myc plasmids were constructed by Genomeditech (Shanghai, China). All the plasmids were verified by DNA sequencing. Transient transfection of HEK293T cells and primary neurons with plasmids was accomplished using Lipofectamine 3000 reagent (Invitrogen, Waltham, MA).

### Coimmunoprecipitation (Co‐IP) and MS

Tissues and cells were lysed in Co‐IP lysis buffer (Proteintech, Rosemont, IL) supplemented with a protease inhibitor cocktail (Proteintech, Rosemont, IL) for 30 min on ice. All the lysed products were transferred to a 1.5 mL centrifuge tube and centrifuged at 12 000 rpm for 30 min, after which the supernatant was collected. An appropriate amount of the supernatant was transferred to a new 1.5 mL centrifuge tube, and an appropriate amount of primary antibody was added. The mixture was mixed evenly and incubated at room temperature on a vertical rotating mixer for 1 h. The remaining supernatant was used as input. After the incubation, pretreated magnetic beads were added to capture the antibody‐antigen complex, and the magnetic beads were collected with the help of a magnet. The collected magnetic beads were washed three times with 0.5% PBST, after which the antigens were eluted with 1 × loading buffer.

For the MS analysis, the protein samples obtained by Co‐IP were electrophoretically separated on an SDS‐PAGE gel. After electrophoresis, the gel was stained with Coomassie blue to observe the different bands. A rubber shovel was used to select the target area of the gel, which was subsequently sent to OE Biotech for further analysis.

### Molecular Docking

AlphaFold2 was used to predict the crystal structures corresponding to YBX1 and TRIM56. The obtained protein crystal structures were processed using the Protein Preparation Wizard module of Schrödinger software to perform protein preprocessing, regenerate the states of the native ligand, optimize H‐bond assignments, minimize protein energy, and remove water. The protein–protein interaction simulation was performed on the processed protein (using the protein–protein docking module), the number of ligand rotations was set to 70 000, and the maximum number of poses was set to 30. The different chains of the most stable protein–protein interaction complex were marked with different colors, and a surface was added to display a 3D view. In addition, the protein interaction analysis module was used to determine the specific segment where YBX1 binds to TRIM56.

### Oxygen Glucose Deprivation (OGD) Treatment

In this study, an OGD model was constructed to simulate SCI in vitro. Briefly, Neurobasal medium was replaced with sugar‐free medium for primary neuron culture. The dish in which the neurons were cultured was transferred to a hypoxic incubator, and the parameters were set to 37 °C, 5% CO_2_, and 1% O_2_. After 2 h of incubation, the cells were collected for further analysis.

### Statistical Analysis

This study used SPSS version 25.0 for the statistical analysis of the experimental data. The data were presented as the means ± standard errors of the means (SEMs). Normalization was performed for the data presented in this study to control for unwanted sources of variation. Comparisons between two independent groups were performed using a two‐tailed, unpaired t test. Two‐way ANOVA combined with Tukey's multiple comparison test was used to determine differences among three or more groups. *p* < 0.05 was considered to indicate statistical significance. ns indicates that the difference was not significant. The symbol * indicates *p* < 0.05.

## Conflict of Interest

The authors declare no conflict of interest.

## Author Contributions

J.L., Y.M., and W.J. contributed equally to this work. J.L., K.Z., M.J., and J.W. designed this study. J.L., Y.M., and W.J. conducted the experiments, analyzed the data and wrote the manuscript. H.S., Y.F., Q.Y., C.Z., and Z.W. analyzed and checked the data. J.L., K.Z., M.J., and J.W. revised the manuscript. All authors read and approved the manuscript prior to submission.

## Supporting information



Supporting Information

## Data Availability

The data that support the findings of this study are available from the corresponding author upon reasonable request.

## References

[1] P. G. Slater , M. E. Dominguez‐Romero , M. Villarreal , V. Eisner , J. Larrain , Cell. Mol. Life Sci. 2022, 79, 239. 35416520 10.1007/s00018-022-04261-x PMC11072423

[2] C. S. Ahuja , J. R. Wilson , S. Nori , M. R. N. Kotter , C. Druschel , A. Curt , M. G. Fehlings , Nat. Rev. Dis. Primers 2017, 3, 17018. 28447605 10.1038/nrdp.2017.18

[3] A. Fang , Y. Wang , N. Guan , Y. Zuo , L. Lin , B. Guo , A. Mo , Y. Wu , X. Lin , W. Cai , X. Chen , J. Ye , Z. Abdelrahman , X. Li , H. Zheng , Z. Wu , S. Jin , K. Xu , Y. Huang , X. Gu , B. Yu , X. Wang , Nat. Commun. 2023, 14, 4011. 37419902 10.1038/s41467-023-39745-2 PMC10328956

[4] S. A.‐O. Eming , T. A.‐O. Wynn , P. A.‐O. Martin , Science 2017, 356, 1026. 28596335 10.1126/science.aam7928

[5] D. J. Hellenbrand , C. M. Quinn , Z. J. Piper , C. N. Morehouse , J. A. Fixel , A. S. Hanna , J. Neuroinflammation 2021, 18, 284. 34876174 10.1186/s12974-021-02337-2 PMC8653609

[6] a) B. Xu , Z. Zhou , J. Fang , J. Wang , K. Tao , J. Liu , S. Liu , Free Radic Biol. Med. 2023, 208, 319; 37640169 10.1016/j.freeradbiomed.2023.08.026

[7] A. Pandeya , T.‐D. Kanneganti , Trends Mol. Med. 2024, 30, 74. 37977994 10.1016/j.molmed.2023.10.001 PMC10842719

[8] L. Wang , Y. Zhu , L. Zhang , L. Guo , X. Wang , Z. Pan , X. Jiang , F. Wu , G. He , Cell Death Dis. 2023, 14, 851. 38129399 10.1038/s41419-023-06370-2 PMC10739961

[9] Q. Xiang , Z. X. Geng , X. Yi , X. Wei , X. H. Zhu , D. S. Jiang , Trends Pharmacol. Sci. 2024, 45, 739. 39003157 10.1016/j.tips.2024.06.002

[10] J. Tong , X. T. Lan , Z. Zhang , Y. Liu , D. Y. Sun , X. J. Wang , S. X. Ou‐Yang , C. L. Zhuang , F. M. Shen , P. Wang , D. J. Li , Acta Pharmacol. Sin. 2023, 44, 1014. 36323829 10.1038/s41401-022-01010-5 PMC10104837

[11] D. K. W. Ocansey , F. Qian , P. Cai , S. Ocansey , S. Amoah , Y. Qian , F. Mao , Theranostics 2024, 14, 640. 38169587 10.7150/thno.91814 PMC10758053

[12] a) M. Feng , X. Xie , G. Han , T. Zhang , Y. Li , Y. Li , R. Yin , Q. Wang , T. Zhang , P. Wang , J. Hu , Y. Cheng , Z. Gao , J. Wang , J. Chang , M. Cui , K. Gao , J. Chai , W. Liu , C. Guo , S. Li , L. Liu , F. A.‐O. Zhou , J. Chen , H. Zhang , Blood 2021, 1, 71; 10.1182/blood.2020009676 PMC8667054 33763698

[13] Y. Xiao , G. P. Cai , X. Feng , Y. J. Li , W. H. Guo , Q. Guo , Y. Huang , T. Su , C. J. Li , X. H. Luo , Y. J. Zheng , M. Yang , EMBO J. 2023, 42, 111762. 10.15252/embj.2022111762 PMC10152142 36943004

[14] S. Chen , K. Li , J. Guo , H. N. Chen , Y. Ming , Y. Jin , F. Xu , T. Zhang , Y. Yang , Z. Ye , W. Liu , H. Ma , J. Cheng , J. K. Zhou , Z. Li , S. Shen , L. Dai , Z. G. Zhou , H. Xu , Y. Peng , Proc. Natl. Acad. Sci. 2023, 120, e2215132120. 36961927 10.1073/pnas.2215132120 PMC10068820

[15] a) B. Ye , H. Zhou , Y. Chen , W. Luo , W. Lin , Y. Zhao , J. Han , X. Han , W. Huang , G. Wu , X. Wang , G. Liang , Circ. Res. 2023, 132, 465; 36722348 10.1161/CIRCRESAHA.122.321849

[16] S. Xu , X. Wu , S. Wang , M. Xu , T. Fang , X. Ma , M. Chen , J. Fu , J. Guo , S. Tian , T. Tian , X. Cheng , H. Yang , J. Zhou , Z. Wang , Y. Yin , W. Xu , F. Xu , J. Yan , Z. Wang , S. Luo , X. J. Zhang , Y. X. Ji , J. Weng , J. Clin. Invest. 2024, 134, e166149. 38206764 10.1172/JCI166149 PMC10904058

[17] a) C. Yang , Z. Wang , Y. Kang , Q. Yi , T. Wang , Y. Bai , Y. Liu , Autophagy 2023, 19, 1934; 36692217 10.1080/15548627.2022.2164427 PMC10283440

[18] J. Lou , M. Jin , C. Zhou , Y. Fan , L. Ni , Y. Mao , H. Shen , J. Li , H. Zhang , C. Fu , X. Mao , Y. Chen , J. Zhong , K. Zhou , L. Wang , J. Wu , Free Radic Biol. Med. 2024, 212, 133. 38142951 10.1016/j.freeradbiomed.2023.12.020

[19] R. A.‐O. Karki , S. A.‐O. X. Lee , R. A.‐O. Mall , N. Pandian , Y. A.‐O. Wang , B. A.‐O. X. Sharma , R. A.‐O. Malireddi , D. A.‐O. Yang , S. A.‐O. Trifkovic , J. A.‐O. Steele , J. A.‐O. Connelly , G. Vishwanath , M. A.‐O. Sasikala , D. N. Reddy , P. A.‐O. Vogel , S. A.‐O. X. Pruett‐Miller , R. A.‐O. Webby , C. A.‐O. Jonsson , T. A.‐O. Kanneganti , Sci Immunol. 2022, 7, eabo6294. 35587515 10.1126/sciimmunol.abo6294 PMC9161373

[20] a) K. Zhou , Z. Zheng , Y. Li , W. Han , J. Zhang , Y. Mao , H. Chen , W. Zhang , M. Liu , L. Xie , H. Zhang , H. Xu , J. Xiao , Theranostics 2020, 10, 9280; 32802192 10.7150/thno.46566 PMC7415792

[21] S. Miller , B. Tavshanjian , A. Oleksy , O. Perisic , B. T. Houseman , K. M. Shokat , R. L. Williams , Science 2010, 327, 1638. 20339072 10.1126/science.1184429 PMC2860105

[22] S. R. Bonam , S. Muller , J. Bayry , D. J. Klionsky , Autophagy 2020, 16, 2260. 32522067 10.1080/15548627.2020.1779467 PMC7755324

[23] C. A.‐O. Zheng , Y. A.‐O. Wei , Q. A.‐O. Zhang , M. A.‐O. Sun , Y. A.‐O. Wang , J. A.‐O. Hou , P. A.‐O. Zhang , X. A.‐O. Lv , D. A.‐O. Su , Y. Jiang , J. A.‐O. Gumin , N. A.‐O. Sahni , B. A.‐O. Hu , W. A.‐O. Wang , X. A.‐O. Chen , D. A.‐O. McGrail , C. Zhang , S. A.‐O. Huang , H. A.‐O. X. Xu , J. A.‐O. Chen , F. A.‐O. X. Lang , J. A.‐O. Hu , Y. A.‐O. Chen , Sci. Adv. 2023, 9, eadf3984. 37540752 10.1126/sciadv.adf3984 PMC10403220

[24] Q. Chen , H. Wang , Z. Li , F. Li , L. Liang , Y. Zou , H. Shen , J. Li , Y. Xia , Z. Cheng , T. Yang , K. Wang , F. Shen , J Hepatol 2022, 76, 135. 34509526 10.1016/j.jhep.2021.08.027

[25] L. Liu , Y. Chen , T. Zhang , G. Cui , W. Wang , G. Zhang , J. Li , Y. Zhang , Y. Wang , Y. Zou , Z. Ren , W. Xue , R. Sun , Adv. Sci. (Weinh) 2024, 11, e2302379. 38566431 10.1002/advs.202302379 PMC11132058

[26] a) R. Wu , S. Cao , F. Li , S. Feng , G. Shu , L. Wang , P. Gao , X. Zhu , C. Zhu , S. Wang , Q. Jiang , FASEB J. 2022, 36, e22219; 35195911 10.1096/fj.202101810RR

[27] R. Wu , S. Feng , F. Li , G. Shu , L. Wang , P. Gao , X. Zhu , C. Zhu , S. Wang , Q. Jiang , Cell Death Dis. 2023, 14, 29. 36642732 10.1038/s41419-023-05564-y PMC9841012

[28] Z. Shi , S. Yuan , L. Shi , J. Li , G. Ning , X. Kong , S. Feng , Cell Prolif. 2021, 54, e12992. 33506613 10.1111/cpr.12992 PMC7941236

[29] a) J. F. Lin , P. S. Hu , Y. Y. Wang , Y. T. Tan , K. Yu , K. Liao , Q. N. Wu , T. Li , Q. Meng , J. Z. Lin , Z. X. Liu , H. Y. Pu , H. Q. Ju , R. H. Xu , M. Z. Qiu , Signal Transduct Target Ther. 2022, 7, 54; 35221331 10.1038/s41392-022-00889-0 PMC8882671

[30] a) S. Christgen , R. E. Tweedell , T. D. Kanneganti , Pharmacol. Ther. 2022, 232, 108010; 34619283 10.1016/j.pharmthera.2021.108010 PMC8930427

[31] M. Bai , Y. Cui , Z. Sang , S. Gao , H. Zhao , X. Mei , Free Rad. Biol. Med. 2024, 221, 169. 38782079 10.1016/j.freeradbiomed.2024.05.037

[32] a) R. Karki , B. Sundaram , B. R. Sharma , S. Lee , R. K. S. Malireddi , L. N. Nguyen , S. Christgen , M. Zheng , Y. Wang , P. Samir , G. Neale , P. Vogel , T. D. Kanneganti , Cell Rep. 2021, 37, 109858; 34686350 10.1016/j.celrep.2021.109858 PMC8853634

[33] a) H. Lei , H. Xu , L. Yang , Y. Wang , Y. Zhang , Y. Wu , Oncogene 2024, 43, 539; 38104157 10.1038/s41388-023-02921-1

[34] Q. Zhang , J. Zheng , W. Wu , H. Lian , N. Iranzad , E. Wang , L. Yang , X. Wang , X. Jiang , Cell Death Dis. 2023, 14, 178. 36870986 10.1038/s41419-023-05702-6 PMC9985612

[35] G. J. Seo , C. Kim , W. J. Shin , E. H. Sklan , H. Eoh , J. U. Jung , Nat. Commun. 2018, 9, 613. 29426904 10.1038/s41467-018-02936-3 PMC5807518

[36] Y. Liu , Y. Chen , C. Ding , X. Zhu , X. Song , Y. Ren , Q. Wang , Y. Zhang , X. Sun , Int. J. Biol. Macromol. 2022, 219, 571. 35952808 10.1016/j.ijbiomac.2022.08.019

[37] a) Q. Hu , J. Xu , L. Wang , Y. Yuan , R. Luo , M. Gan , K. Wang , T. Zhao , Y. Wang , T. Han , J. B. Wang , Adv. Sci. (Weinh) 2023, 10, e2303535; 37904651 10.1002/advs.202303535 PMC10724390

[38] a) R. Verbeke , M. J. Hogan , K. Lore , N. Pardi , Immunity 2022, 55, 1993; 36351374 10.1016/j.immuni.2022.10.014 PMC9641982

[39] J. A.‐O. X. Squair , M. A.‐O. Milano , A. A.‐O. de Coucy , M. A.‐O. Gautier , M. A.‐O. Skinnider , N. A.‐O. James , N. A.‐O. X. Cho , A. Lasne , C. A.‐O. Kathe , T. A.‐O. Hutson , S. A.‐O. Ceto , L. A.‐O. Baud , K. A.‐O. Galan , V. A.‐O. X. Aureli , A. A.‐O. Laskaratos , Q. A.‐O. Barraud , T. A.‐O. Deming , R. A.‐O. X. Kohman , B. A.‐O. Schneider , Z. A.‐O. He , J. A.‐O. Bloch , M. A.‐O. Sofroniew , G. A.‐O. Courtine , M. A.‐O. Anderson , Science 2023, 381, 1338. 37733871 10.1126/science.adi6412

[40] a) S. Li , Y. Sun , M. Song , Y. Song , Y. Fang , Q. Zhang , X. Li , N. Song , J. Ding , M. Lu , G. Hu , JCI Insight 2021, 6, 1993; 10.1172/jci.insight.146852 PMC8675200 34877938

