# Supplementary material for: TRIM56 Modulates YBX1 Degradation to Ameliorate ZBP1‐Mediated Neuronal PANoptosis in Spinal Cord Injury
Source: Adv Sci (Weinh). 2024 Sep 18;11(42):2407132. doi: 10.1002/advs.202407132 (PMC11558135; doi:10.1002/advs.202407132)
Supplement: Supplementary file 1 — Supporting Information [file ADVS-11-2407132-s001.pdf]

ADVANCED SCIENCE
Open Access

Supporting Information

for *Adv. Sci.*, DOI 10.1002/advs.202407132

TRIM56 Modulates YBX1 Degradation to Ameliorate ZBP1-Mediated Neuronal PANoptosis in Spinal Cord Injury

Junsheng Lou, Yiting Mao, Wu Jiang, Honghao Shen, Yunpeng Fan, Qing Yu, Conghui Zhou, Ziyao Wei, Kailiang Zhou, Mengran Jin* and Junsong Wu**

Supporting Information

TRIM56 Modulates YBX1 Degradation to Ameliorate ZBP1-mediated Neuronal PANoptosis in Spinal Cord Injury

Junsheng Lou#, Yiting Mao#, Wu Jiang#, Honghao Shen, Yunpeng Fan, Qing Yu, Conghui Zhou, Ziyao Wei, Kailiang Zhou*, Mengran Jin*, Junsong Wu*

Supplemental Table 1. Primer sequences for qPCR analysis.

Gene	Species	Forward primer	Reverse primer
Ybx1	Mouse	CAGACCGTAACCATTATAGACGC	ATCCCTCGTTCTTTTCCCCAC
Zbp1	Mouse	AAGAGTCCCCTGCGATTATTTG	TCTGGATGGCGTTTGAATTGG
Trim56	Mouse	AAGACTCCTCCCCAACTCTG	GGCAATAGGTATGTAGGCATGG
β-actin	Mouse	GGCTCCTAGCACCATGAAGA	AGCTCAGTAACAGTCCGCC
Ybx1	Human	TAGACGCTATCCACGTCGTAG	ATCCCTCGTTCTTTTCCCCAC
β-actin	Human	CATGTACGTTGCTATCCAGGC	CTCCTTAATGTCACGCACGAT

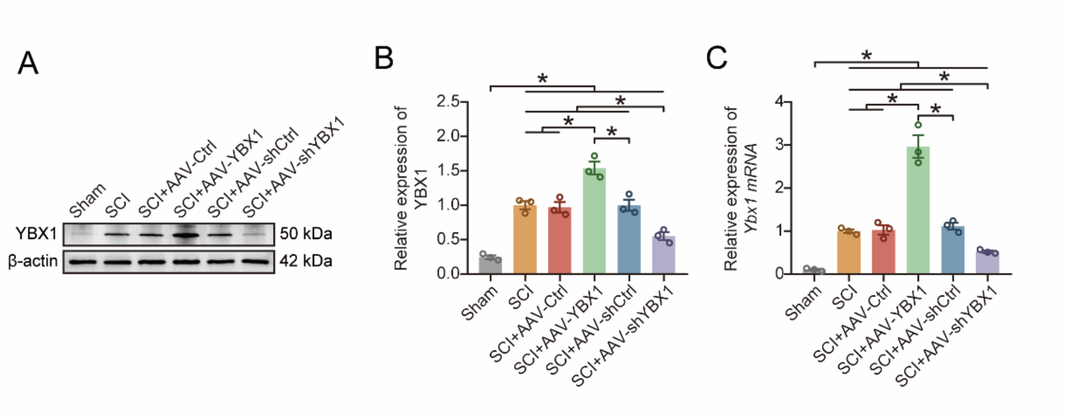

Supplemental Figure 1. Validation of YBX1 overexpression efficiency and knockdown efficiency. (A, B) Western blot and quantification of YBX1 expression in spinal cord tissue on day 3 after SCI (n = 3). **(C)** qPCR analysis of *Ybx1* abundance in spinal cord tissue on day 3 after SCI (n = 3). The data are presented as the means ± SEMs; *$p < 0.05$. Significance was calculated using two-way ANOVA combined with Tukey's multiple comparison test.

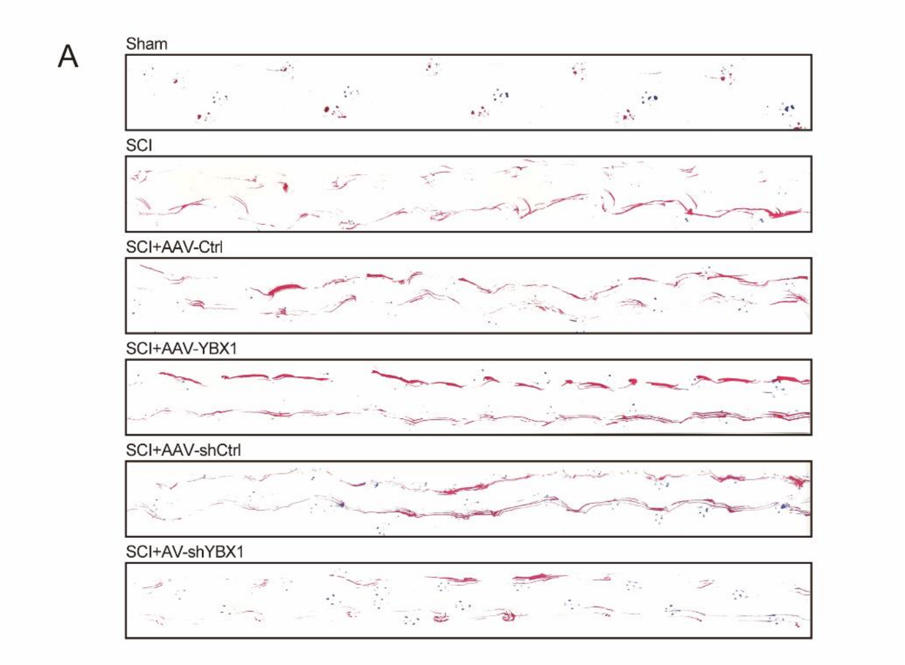

Supplemental Figure 2. (A) Scans of footprint test results on day 28 after SCI (n = 10).

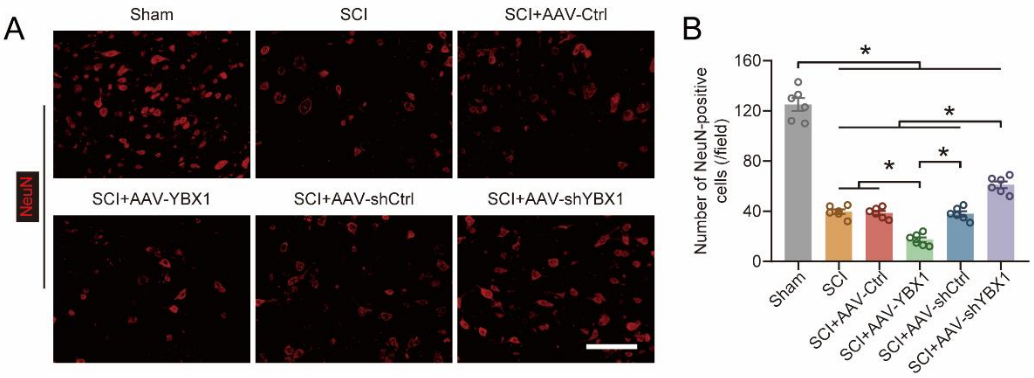

Supplemental Figure 3. Detection of surviving neurons. (A, B) Representative immunohistochemical images of NeuN^{+} neurons in injured spinal cord of mice on day 28 after SCI (n = 6; scale bar: 50 μm). The data are presented as the means ± SEMs; **p* < 0.05. Significance was calculated using two-way ANOVA combined with Tukey's multiple comparison test.

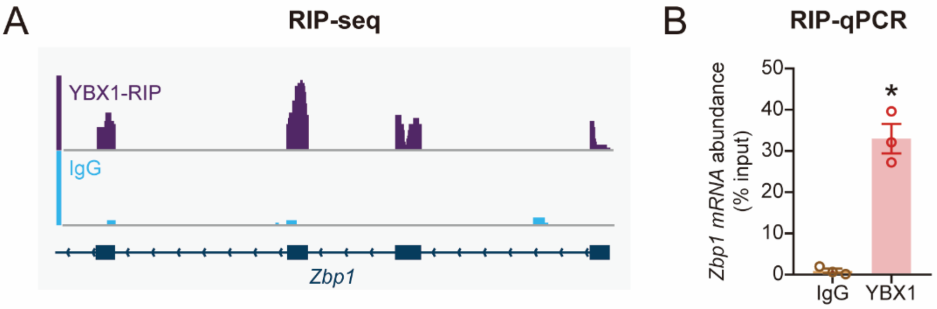

Supplemental Figure 4. Detection of the binding of YBX1 to Zbp1 transcripts. (A) An integrative genomics viewer displaying the enrichment of *Zbp1* transcripts after RIP. **(B)** YBX1 RIP-qPCR detects the binding between YBX1 and *Zbp1* transcripts (n = 3). The data are presented as the means ± SEMs; **p* < 0.05. Significance was calculated using a two-tailed, unpaired t test.

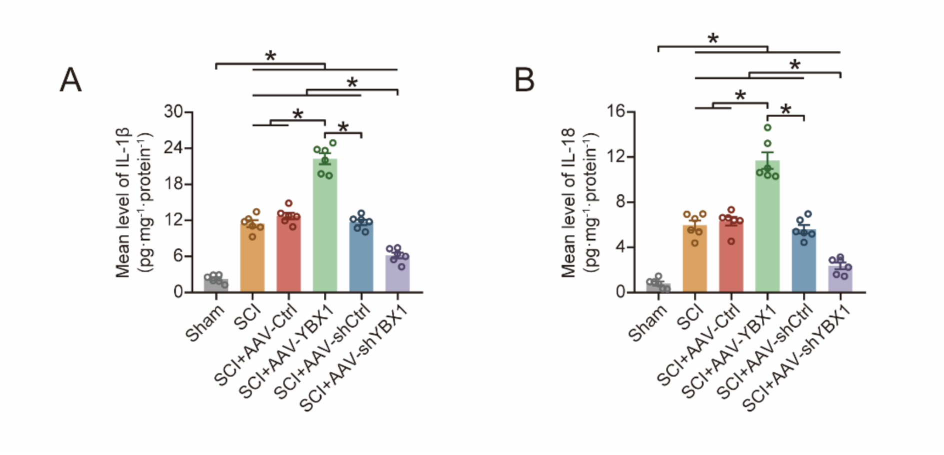

Supplemental Figure 5. Detection of PANoptosis-related inflammatory factors. (A, B) Determination of mean levels of IL-18 and IL-1β in injured spinal cord by ELISA kit (n = 6). The data are presented as the means ± SEMs; *$p < 0.05$. Significance was calculated using two-way ANOVA combined with Tukey's multiple comparison test.

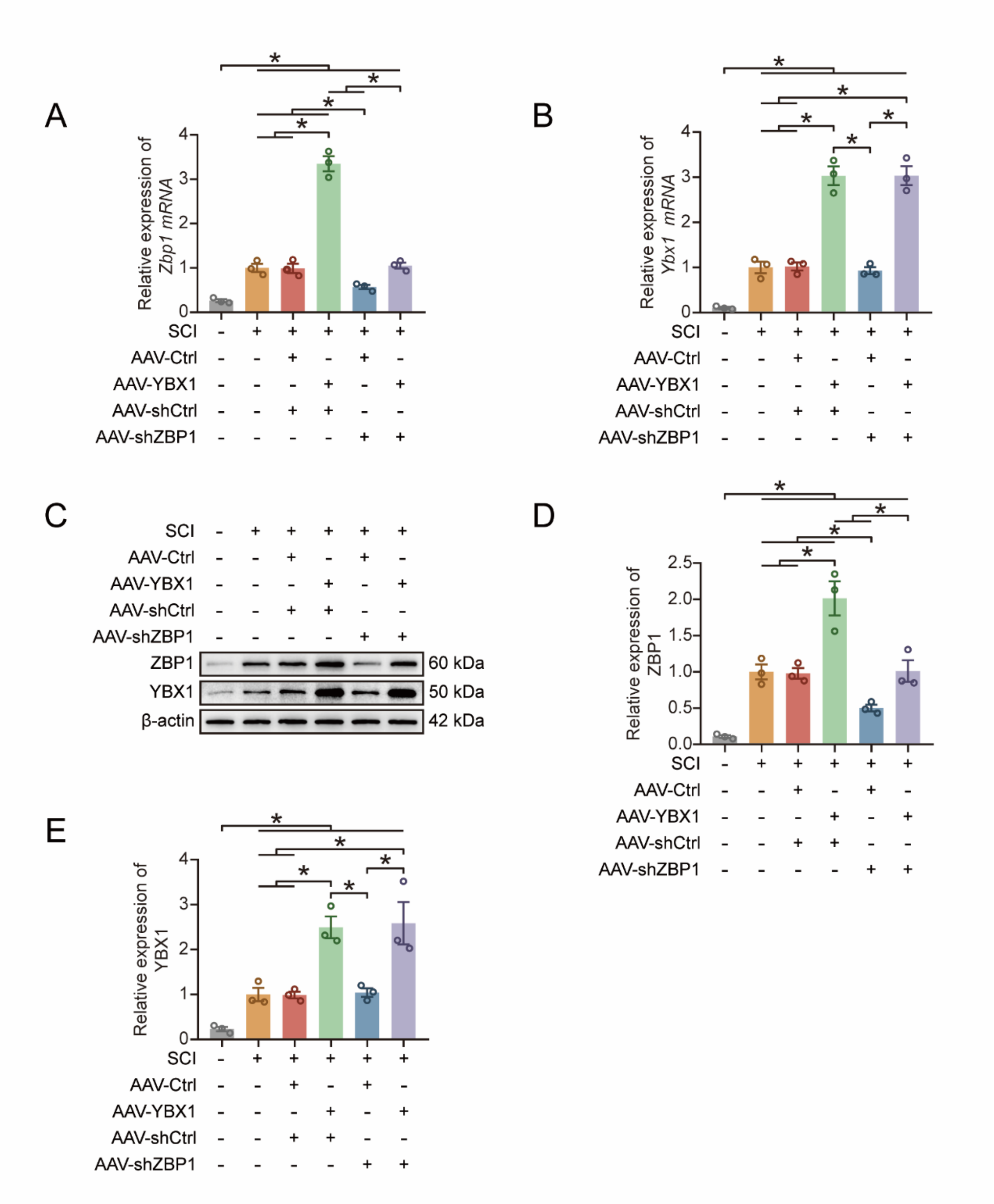

Supplemental Figure 6. Validation of YBX1 overexpression efficiency and ZBP1 knockdown efficiency. (A, B) qPCR analysis of *Zbp1* and *Ybx1* abundance in spinal cord tissue on day 3 after SCI (n = 3). **(C-E)** Western blot and quantification of ZBP1 and YBX1 expression in spinal cord tissue on day 3 after SCI (n = 3). The data are presented as the means ± SEMs; $*p < 0.05$. Significance was calculated using two-way ANOVA combined with Tukey's multiple comparison test.

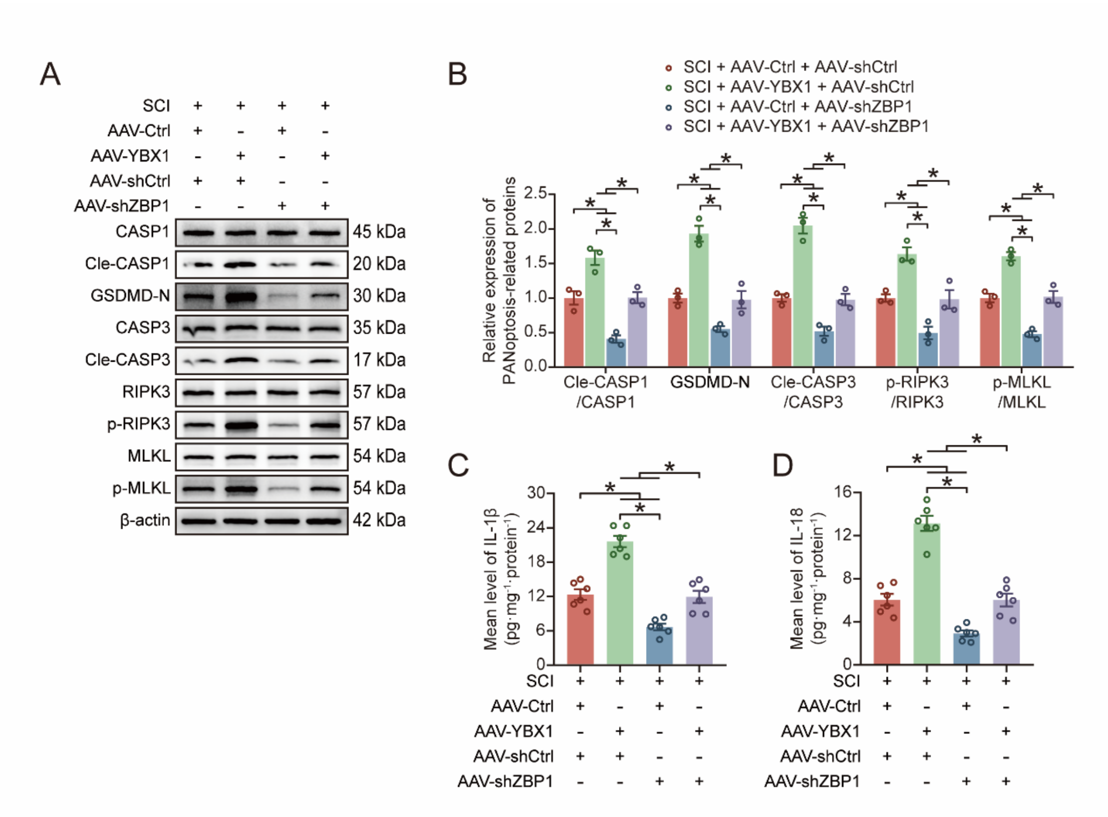

Supplemental Figure 7. YBX1 regulates PANoptosis after SCI by mediating the stability of *Zbp1*. (A, B) Western blot and quantification of PANoptosis-related protein expression in spinal cord tissue on day 3 after SCI (n = 3). **(C, D)** Determination of mean levels of IL-18 and IL-1β in injured spinal cord by ELISA kit (n = 6). The data are presented as the means ± SEMs; *$p < 0.05$. Significance was calculated using two-way ANOVA combined with Tukey's multiple comparison test.

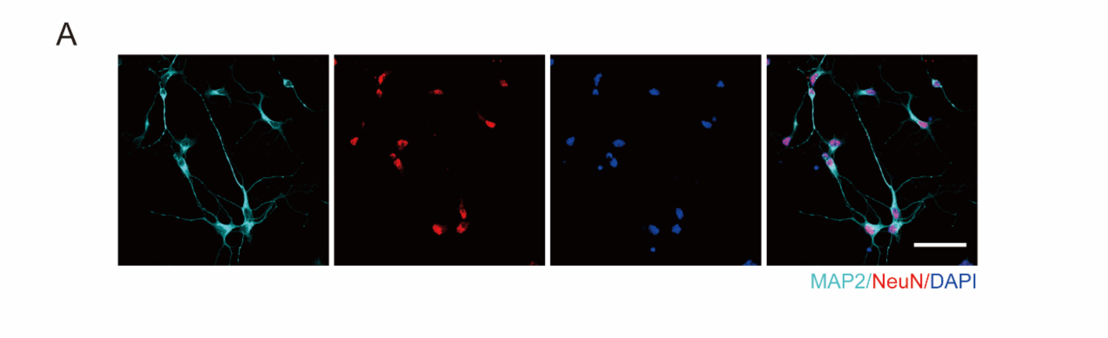

Supplemental Figure 8. (A) The extracted primary neurons were verified using specific markers MAP2 and NeuN (Scale bar: 40 μm).

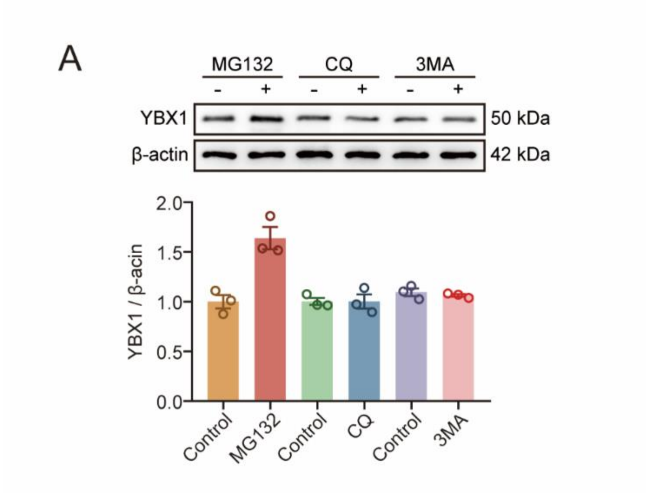

Supplemental Figure 9. (A) Western blot and quantification of YBX1 expression in Primary neurons (n = 3). Primary neurons were pretreated with MG132 (10 μM), 3-MA (10 mM), or CQ (25 μM) for 6 h before harvest. The data are presented as the means ± SEMs; *$p < 0.05$. Significance was calculated using two-way ANOVA combined with Tukey's multiple comparison test.

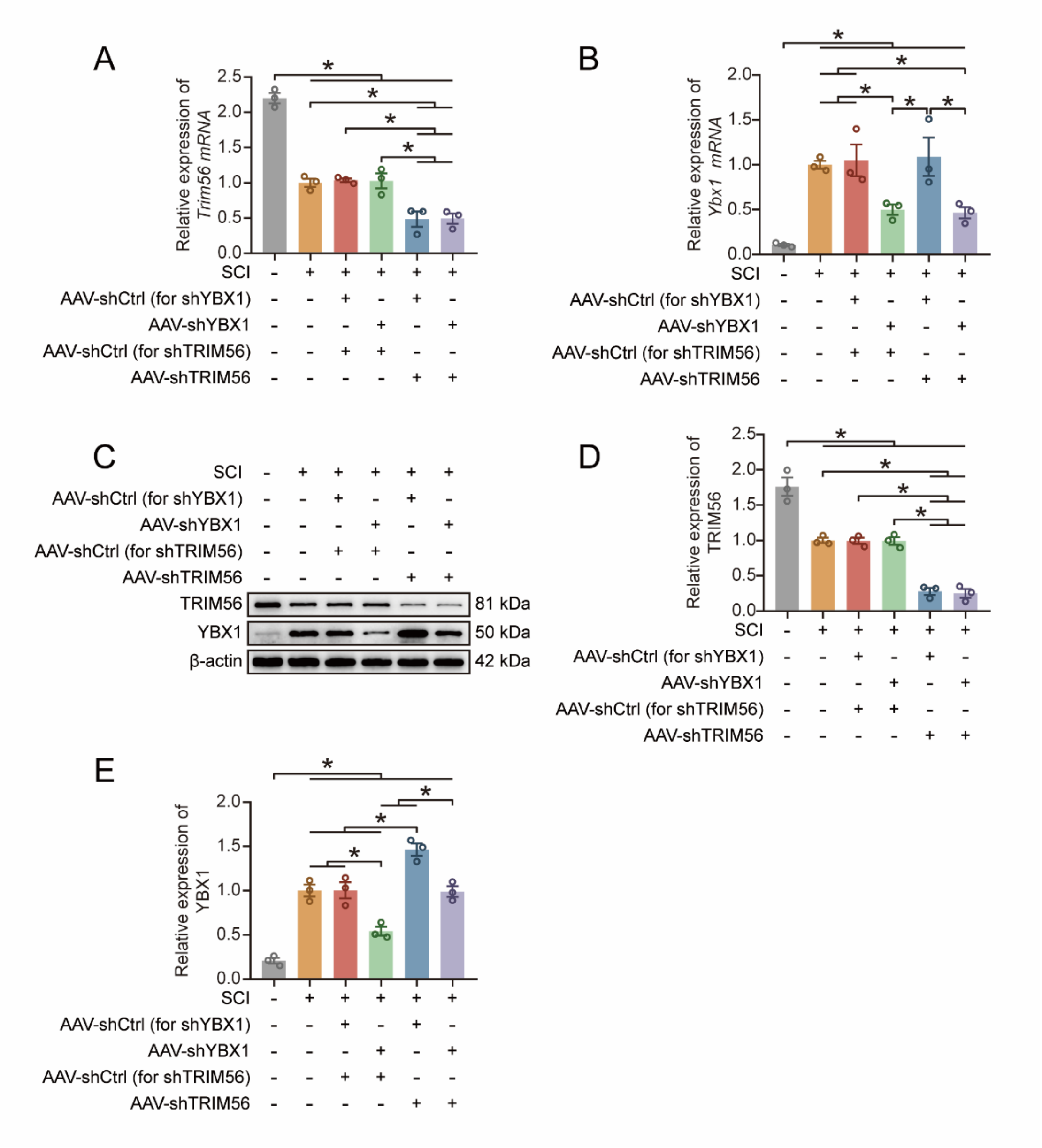

Supplemental Figure 10. Validation of TRIM56 and YBX1 knockdown efficiency. **(A, B)** qPCR analysis of *Trim56* and *Ybx1* abundance in spinal cord tissue on day 3 after SCI (n = 3). **(C-E)** Western blot and quantification of TRIM56 and YBX1 expression in spinal cord tissue on day 3 after SCI (n = 3). The data are presented as the means ± SEMs; *$p < 0.05$. Significance was calculated using two-way ANOVA combined with Tukey's multiple comparison test.

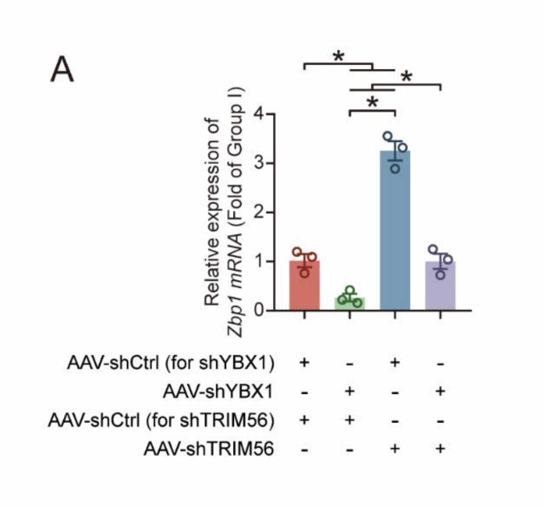

Supplemental Figure 11. (A) qPCR analysis of *Zbp1* abundance in spinal cord tissue on day 3 after SCI (n = 3). The data are presented as the means ± SEMs; *$p < 0.05$. Significance was calculated using two-way ANOVA combined with Tukey's multiple comparison test.

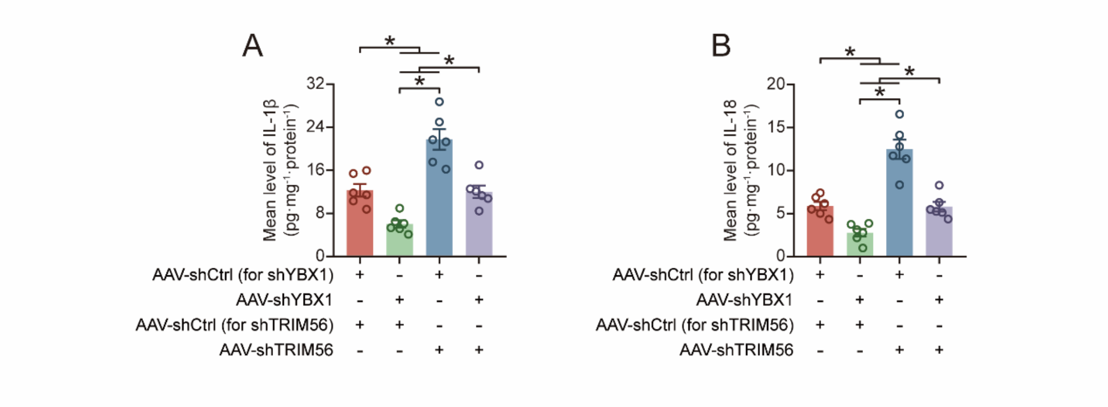

Supplemental Figure 12. Detection of PANoptosis-related inflammatory factors. (A, B) Determination of mean levels of IL-18 and IL-1β in injured spinal cord by ELISA kit (n = 6). The data are presented as the means ± SEMs; *$p < 0.05$. Significance was calculated using two-way ANOVA combined with Tukey's multiple comparison test.

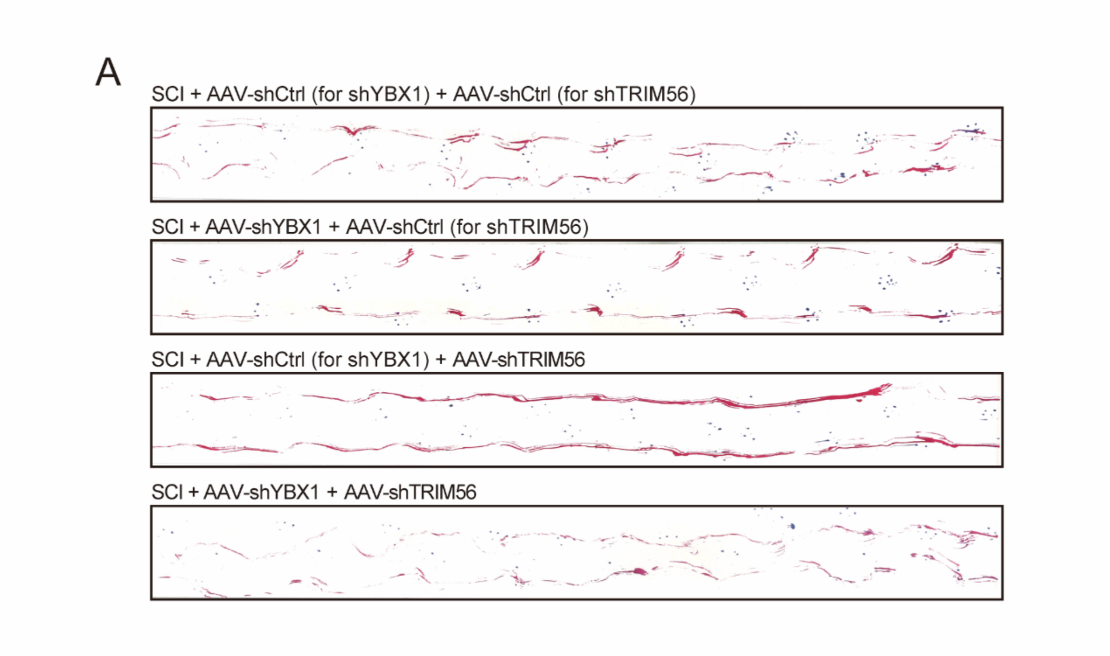

Supplemental Figure 13. (A) Scans of footprint test results on day 28 after SCI (n = 10).

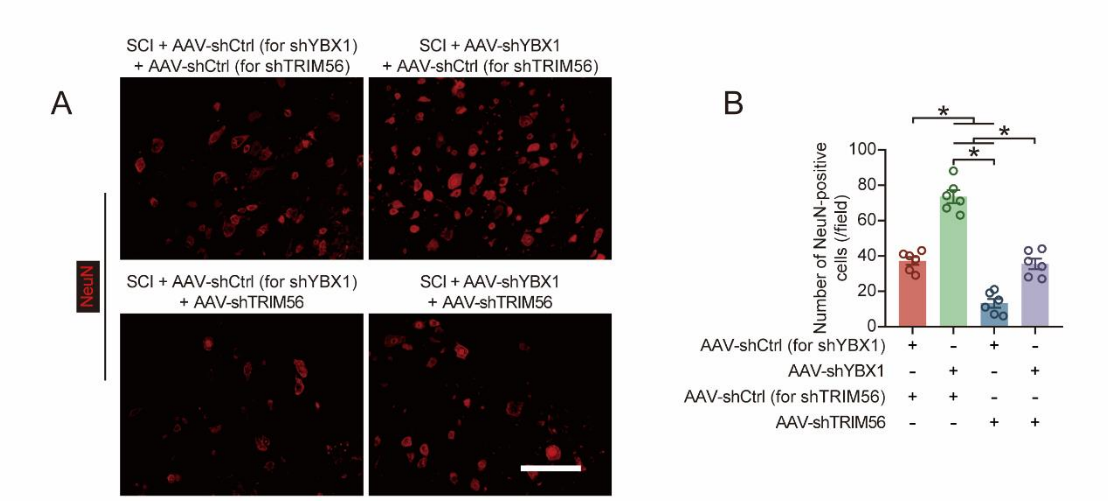

Supplemental Figure 14. Detection of surviving neurons. (A, B) Representative immunohistochemical images of $NeuN^+$ neurons in injured spinal cord of mice on day 28 after SCI (n = 6; scale bar: 50 μm). The data are presented as the means ± SEMs; **p* < 0.05. Significance was calculated using two-way ANOVA combined with Tukey's multiple comparison test.
